# An overview of carbapenem-resistant organisms from food-producing animals, seafood, aquaculture, companion animals, and wildlife

**DOI:** 10.3389/fvets.2023.1158588

**Published:** 2023-06-15

**Authors:** Flor Y. Ramírez-Castillo, Alma L. Guerrero-Barrera, Francisco J. Avelar-González

**Affiliations:** ^1^Laboratorio de Biología Celular y Tisular, Departamento de Morfología, Centro de Ciencias Básicas, Universidad Autónoma de Aguascalientes, Aguascalientes, Ags, Mexico; ^2^Laboratorio de Estudios Ambientales, Departamento de Fisiología y Farmacología, Centro de Ciencias Básicas, Universidad Autónoma de Aguascalientes, Aguascalientes, Ags, Mexico

**Keywords:** carbapenem resistance, One Health approach, food-producing animals, carbapenemase producers, transmission

## Abstract

Carbapenem resistance (CR) is a major global health concern. CR is a growing challenge in clinical settings due to its rapid dissemination and low treatment options. The characterization of its molecular mechanisms and epidemiology are highly studied. Nevertheless, little is known about the spread of CR in food-producing animals, seafood, aquaculture, wildlife, their environment, or the health risks associated with CR in humans. In this review, we discuss the detection of carbapenem-resistant organisms and their mechanisms of action in pigs, cattle, poultry, seafood products, companion animals, and wildlife. We also pointed out the One Health approach as a strategy to attempt the emergency and dispersion of carbapenem-resistance in this sector and to determine the role of carbapenem-producing bacteria in animals among human public health risk. A higher occurrence of carbapenem enzymes in poultry and swine has been previously reported. Studies related to poultry have highlighted *P. mirabilis, E. coli*, and *K. pneumoniae* as NDM-5- and NDM-1-producing bacteria, which lead to carbapenem resistance. OXA-181, IMP-27, and VIM-1 have also been detected in pigs. Carbapenem resistance is rare in cattle. However, OXA- and NDM-producing bacteria, mainly *E. coli* and *A. baumannii*, are cattle's leading causes of carbapenem resistance. A high prevalence of carbapenem enzymes has been reported in wildlife and companion animals, suggesting their role in the cross-species transmission of carbapenem-resistant genes. Antibiotic-resistant organisms in aquatic environments should be considered because they may act as reservoirs for carbapenem-resistant genes. It is urgent to implement the One Health approach worldwide to make an effort to contain the dissemination of carbapenem resistance.

## 1. Introduction

Carbapenems are broad-spectrum beta (**β**)-lactam antimicrobials primarily used to treat severe human infections. These antibiotics are considered one of the most reliable drugs and the last line of therapy for infections caused by multidrug-resistant Gram-negative and Gram-positive bacteria. Carbapenems possess a broad-spectrum antibacterial activity and have a structure defined by a carbapenem coupled with a **β**-lactam ring. In addition, these antibiotics contain a carbon instead of a sulfone in the fourth position of the thiazolidine moiety **β**-lactam ring, which confers protection against most **β**-lactamases (1).

The widespread use of these antibiotics has increased to a worldwide emergence of carbapenem-resistant organisms (CROs), which constitute a critical growing public health threat, mainly in hospital settings, as their prescription has escalated in recent years and used for treating life-threatening infections. Carbapenem-resistant *Enterobacteriaceae* (CRE) [i.e., Carbapenem-resistant *Klebsiella pneumoniae* (CR-Kp), Carbapenem-resistant *Escherichia coli* (CREc), *Enterobacter* spp., *Serratia* spp., and *Proteus* spp.] are some of the most critical CROs because they are associated with infections that lead to high mortality and have the potential to spread carbapenem resistance via mobile genetic elements (2). In addition, non-fermenting bacteria such as carbapenem-resistant *Acinetobacter baumannii* (CRAB) and carbapenem-resistant *Pseudomonas aeruginosa* (CRPA) have also emerged as critical CROs (3–5).

The four significant carbapenem mechanisms of resistance include the presence of **β**-lactamase enzymes called carbapenemases, which hydrolyze carbapenem antibiotics encoded on chromosomal or plasmid genes, the synergistic effect of other **β**-lactamases with bacterial cell membrane permeability due to alterations or mutations in porins, the low affinity of penicillin-binding proteins (PBPs) in different species, and the increased efflux pumps (6, 7).

Among carbapenem-producing (CP) microorganisms, different classes of carbapenemases are found under the Ambler classification (Table 1). Class A or serine carbapenemases can hydrolyze all **β**-lactams, carbapenems, cephalosporins, penicillins, and aztreonam but are inhibited by clavulanate and tazobactam. Additionally, a combination of the newly cephalosporin antibiotic, ceftaroline, and avibactam (ceftaroline/avibactam) has been shown to produce activity against *Enterobacteriaceae* KPC producers (19). *Klebsiella pneumoniae* carbapenemase (KPC), not metalloenzyme carbapenemase (NMC-A), imipenem-hydrolyzing beta-lactamase (IMI), and *Serratia marcescens* enzyme (SME) are representative of this class. KPC enzymes confer resistance to all **β**-lactamases and other types of antibiotics, such as quinolones and aminoglycosides. They are only partially inhibited by **β**-lactamase inhibitors such as clavulanic acid, tazobactam, and boronic acid (1–7).

**Table 1:** Carbapenem-enzymes among carbapenem-resistance organisms on Ambler classification.

**Ambler class**		**Class**	**Encoded**		**References**
			**Chromosomal**	**Plasmid**	
Serin carbapenemases	Uses the amino acid serine for beta-lactam hydrolysis by forming an acyl enzyme	A	NmcA (Non-metallo carbapenemase A)	KPC (*K. pneumoniae* carbapenemases), 23 variants	(8–13)
					SME (*Serratia marcescens* enzyme)	GES (Guiana extended spectrum **β**-lactamase), 26 variants	
					IMI (Imipenem-hydrolyzig **β**-lactamase)	IMI 1–6	
					SFC-1 (*Serratia fonticola* carbapenemase)	VCC-1 (from *Vibrio cholerae*)	
					BIC-1 (Bicêtre Carbapenemae)	FRI-1 (from *Enterobacter cloacae*)	
					PenA (penicilinase from *Pseudomonas cepacia*)		
					FPH-1 (from *Francisella philomiragia*)		
					SHV (natural class A β-lactamase of *K. pneumoniae*)		
					KPC (*K. pneumoniae* carbapenemase)	1–3	
					GES (Guiana extended spectrum **β**-lactamase)	20 variants	
				D	OXA	400 enzymes classified in 12 subgroups: (a) OXA-23, (b) OXA-24/40, (c) OXA-48, (d) OXA-51, (e) OXA-58, (f) OXA-134a, (g) OXA-143, (h) OXA-211, (i) OXA-213, (j) OXA-214, (k) OXA-229, and (l) OXA-235, OXA-181 and OXA-497	(9)
		Metallo-carbapenemases	Requires at least one active-site zinc ions to facilitate beta-lactam hydrolysis	B	NDMs (New Delhi metallo-beta-lactamases)	NDMs (New Delhi metallo-**β**-lactamases), 17 variants	(14)
								VIM (Verona integron-encoded metallo-**β**-lactamase), 14 variants	
						IMP-type (Imipenem resistant *Pseudomonas*), 55 variants	
						VMB-1, VMB-2 (*Vibrio* MBL)	(15)
						GIM (German imipenemase)	(13, 14)
						SIM (Seoul imipenase)	(16)
						VAM-1 (*V. alginolyticus* metallo-**β**-lactamase)	(17)
						SPM (São Paulo MBL)	(14)
						DIM-1 (Dutch imipenemase-1)	(18)
						KHM-1 (Kyorin University Hospital MBL-1)	(13)
						TMB (Tripoli MBL-1)	(13)
						FIM (Florence imipenemase)	(18)
						AIM (Adelaide imipenemase)	(18)
						SFH-1 (*Serratia fonticola* carbapenem hydrolase)	(13)
						LMB-1 (Linz metallo-β-lactamase)	(13)

Class B or metallo-**β**-lactamases (MBLs) use a zinc ion (Zn^2+^) to hydrolyze the **β**-lactam ring. They confer resistance to all **β**-lactam antibiotics but are susceptible to aztreonam and **β**-lactam inhibitors such as ethylenediaminetetraacetic acid (EDTA). Most clinically important MBLs belong to the six different families [imipenem (IMP), Verona integron-encoded metallo-**β**-lactamase (VIM), New Delhi metallo-**β**-lactamase (NDM), São Paulo metallo-**β**-lactamases (SPM), German imipenemase (GIM), and Seoul imipenemase (SIM)]. This type of enzyme has been identified in clinically relevant species such as Enterobacterales, *Acinetobacter*, and *P. aeruginosa*. It is commonly expressed from mobile elements such as integrons, plasmids, and transposons (1–7).

Class D serine oxacillinases (OXAs) have been commonly detected worldwide. They have hydrolytic activity against **β**-lactams, high activity against penicillin, and weak activity against extended-spectrum cephalosporins and carbapenems. OXA-23 and OXA-48 are variants widely dispersed globally. OXA-23 is almost strictly restricted to *Acinetobacter* spp., wily OXA-48 was found among *K. pneumoniae* and Enterobacterales (20).

CROs are usually only susceptible to polymyxins (e.g., colistin), fosfomycin, and tigecycline, while colistin resistance in carbapenem-resistance *K. pneumoniae* (CR-Kp) isolates has been recently reported (21–24). Thus, colistin combination therapy is more frequently used to treat drug-resistant bacteria with significantly lower treatment failure rates (24).

Carbapenems such as imipenem, meropenem, ertapenem, doripenem, biapenem, faropenem, and panipenem have been approved for use in human clinical settings (6). However, carbapenems are not licensed in livestock or veterinary fields; therefore, carbapenem resistance is not commonly tested in animals. However, extended-spectrum beta-lactamases (ESBL), including ceftiofur, cefquinome, cefpodoxime, cefoperazone, and cefovecin, are commonly found in this sector. Additionally, ceftiofur, a third-generation cephalosporin, is the main cephalosporin used in veterinary fields and has been approved for treating bacterial infections in food-producing animals (i.e., pneumonia, arthritis, septicemia, meningitis, metritis, and polyserositis) (25). For this reason, these antibiotics could provide selection pressure that favors the expression of carbapenem-resistant (CR) strains.

Indeed, the author's statement in the Scientific Opinion on carbapenem resistance in food-animal ecosystems that “diagnostic isolates of veterinary origin classified as microbiologically resistant to third- and fourth-generation cephalosporins based on epidemiological cutoff values, should be subjected to phenotypic testing for carbapenem resistance and carbapenemase production and subsequent molecular identification and characterization of carbapenemase production genes because they favor the emergence of carbapenem-resistant isolates” (26).

Many studies have reported CROs in livestock, seafood, companion animals, wildlife, and their environments (7, 27–30). As animals have been identified as a relevant source of multidrug-resistant (MDR) bacteria, they can serve as reservoirs for carbapenem-resistant bacteria, and foodborne routes and emission in the environment through the excreta and the subsequent exposure of humans via the environment serve as transmission pathways for carbapenem-resistance genes from animals to humans and vice versa. Furthermore, their incidence may be underestimated because there is usually no surveillance, which dismisses the potential risks to human health. This review aimed to summarize the occurrence and molecular mechanisms of resistance in carbapenem-resistant organisms in food-producing animals, seafood, companion animals, and wildlife to address the importance of antimicrobial resistance surveillance in these sectors, resistance dissemination to the environment and humans, and potential public health risks.

## 2. Carbapenem-resistant from seafood and aquaculture

Carbapenem-resistance genes have been identified in isolates from aquatic environments. Generally, resistance to carbapenems in bacteria from aquatic systems such as *Vibrio* and *Shewanella* spp., as well as Enterobacterales, is mainly mediated by the production of carbapenemases encoded by chromosomal genes or by plasmids. However, carbapenemases are variable, with Class B enzymes and the enzymes described only on aquatic species such as VMB-1 from *Vibrio alginolyticus* (17, 31) and VMB-2 (32) from *Vibrio diabolicus* on shrimps; as well as the Class A, VCC-1 from non-toxigenic *Vibrio cholerae* on shrimp (8).

In 2017, in Canada, Brouwer et al. isolated the *E. cloacae* complex from shrimp (*Litopenaeus vannamei*) originating in India. The isolated had a ST previously described in companion animals in Japan, ST813, and was positive for the *bla*_IMI − 2_ gene, which is located in a plasmid p3443-IMI2, which is closely related to IncFII plasmids and pIMI-6, which was described in an *E. cloacae* complex clinical isolate from Canada and carries the carbapenemase *bla*_IMI − 6_ (33). The same strain displayed the plasmid p3442-FLC-1 that carries the gene encoding a novel class A carbapenemase FLC-1 with close sequence similarity to *bla*_FRI − 1_, previously described in imipenem-resistant *E. cloacae* recovered from a clinical patient in France (33). In 2013, OXA-23-producing *A. baumannii*, on fish *Pagellus acarne* harvested in the Mediterranean Sea in Algeria, was reported. The isolate belonged to the widespread sequence type 2 (ST2)/international clone II and harbored aminoglycoside-modifying enzymes [*aac(6*′*)*-*Ib* and *aac(3*′*)-I* genes] as well as the naturally occurring *bla*_OXA − 51 − like_ gene. However, the isolates differed from human clinical strains previously isolated from France and Algeria (34). In 2010, in Brazil, a high percentage of resistance to imipenem (71.43%) in *E. coli* isolated from aquaculture was detected, including isolates from pond water, shrimp tissues, and pond sediment (35). In 2014 and 2015, the occurrence of VIM-2-producing *Pseudomonas fluorescens* isolated from squid in Canada (imported from South Korea) and OXA-48-producing bacteria in seafood from China and Korea were described on the bacterial species *Stenotrophomonas maltophilia, Myroides odoratimimus, Stenotrophomonas* spp., and *Pseudomonas putida* (36, 37). In 2015, carbapenem-resistant *Enterobacter* spp., derived from imported retail seafood in Canada were detected, including two *Enterobacter cloacae* isolated from shrimp imported from Vietnam harboring *bla*_IMI − 1_; one *Enterobacter aerogenes* harboring *bla*_IMI − 2_ isolated from shrimp imported from Bangladesh, three *E. cloacae* harboring *bla*_IMI − 1_ isolated from clam imported from Vietnam, and two *E. cloacae* harboring *bla*_NDM − 1_, *bla*_TEM_, and *bla*_OXA − 1_ from clam samples from Vietnam (38). *bla*_IMI − 2_ gene was plasmid-mediated; the plasmid contained the IncFII (Yp) replicon, while *bla*_NDM − 1_ plasmid contained IncHI2, IncFIb, and IncFII replicons. Six different sequence types of *E. cloacae* were assigned (ST479, ST373, ST477, ST478, ST411, and ST412). The authors showed that the human-source *E. cloacae* ST373 isolate harboring *bla*_IMI − 1_ shared >75% similarity with the *E. cloacae* IMI-1 positive isolated from clam. In 2016, VCC-1-producing *Vibrio cholerae* isolated from retail shrimp imported from Canada was also identified (8). In 2016, in Italy, one VIM-1 carbapenemase-producing *E. coli* (ST10) was isolated from a Venus's clam (*Ruditapes philippinarum*) harvested in the Mediterranean Sea with *bla*_VIM − 1_ as part of the variable region of a class I integron embedded in a Tn*3*-like transposon that also contained the fluoroquinolone resistance gene *qnrS1*. Interestingly, *E. coli* ST10 is widespread among clinical and animal samples (39). In 2018, six *bla*_NDM_-harboring *Enterobacteriaceae* (four *K. pneumoniae* strains and two *E. coli* strains) from the retail fish market were detected in India, including the variants *bla*_NDM − 5_, *bla*_NDM − 2_, and *bla*_NDM − 1_. The *bla*_NDM_-positive *E. coli* isolates belonged to the multidrug-resistant widespread ST131 clone, representing extra-intestinal pathogenic *E. coli*. ST131 clone is widely distributed among human clinical isolates from urinary tract infections (UTIs). Moreover, they found that all the isolates were resistant to all **β**-lactam antibiotics, quinolones, trimethoprim-sulfamethoxazole, chloramphenicol, and tetracycline (40). In China, several isolates of *Vibrio* spp. were found to be resistant to imipenem and meropenem. Isolates from shrimps of seafood carried the genes *bla*_NDM − 1_, *bla*_VIM − 1_ (31), and *bla*_VMB − 2_ identified on a plasmid-borne composite transposon IS*Shfr9*-IS*CR1*-*bla*_VMB − 2_-*bla*_CARB − 12_-*aadA1*-IS*Shfr9*, where IS*Shfr9* was found to be disseminated in multidrug-resistant (MDR) pathogens (32); as well as producing the enzymes VMB-1 encoded by a gene *bla*_VMB − 1_ located in an integron bearing, highly transmissible IncC type plasmid, namely pVB1796 (15), and VAM-1 located in a conjugative plasmid, namely, pC1579 (17). Other studies in Korea have reported carbapenem-resistant *Vibrio* spp., isolates from shrimp (41), cockles (42), or hard-shell mussels harboring *bla*_OXA_ genes (43). In Europe, in 2018 and 2017, Italy reported *V. cholerae* resistant to meropenem (44) and *V. vulnificus* isolated from shellfish resistant to imipenem and meropenem (45). KPC-3-producing *E. coli* in mussels (*Mytilus galloprovincialis*) and OXA-23-producing *A. baumannii* ST2 isolates from mussels and oysters (*Crassostrea gigas*) were also reported in Tunisia in 2016 (46, 47). The *bla*_KPC − 3_ gene was identified on an ~180 kb IncFII plasmid carrying Tn*4401d* transposon and belonged to the ST167 phylogroup A of the ST10 complex. Interestingly, the authors mention that the predominance of *bla*_KPC − 3_ in Portugal was also associated with the spread of an IncF plasmid carrying Tn*4401d*. The ST10 complex was reported previously in a hospital from the US to spread *bla*_KPC_ genes. In 2018, France reported the isolation of NDM-1-producing *V. parahaemolyticus* ST864 from a shelled shrimp tail imported from Vietnam, which harbored the epidemic plasmid IncA/C (48). In South America and Ecuador in 2015, *Vibrio* spp., resistant to imipenem, was isolated from shrimp from seawater (49). In 2020, *E. coli, Enterobacter cloacae* complex, and *K. pneumoniae* were found in tilapia fish from Egyptian fish farms carrying *bla*_KPC_, *bla*_OXA − 48_, and *bla*_NDM_ (50). In 2018, in Taiwan, carbapenem-resistant *Shewanella algae* were isolated from small abalone (*Haliotis diversicolor*) harboring genes encoding OXA-55 and multiple antibiotic-resistance genes including *dfrA3* (trimethoprim resistance), *tet* (35) (tetracycline resistance), and *qnrA3* (quinolone resistance); and the *pmrCAB* operon, which has been shown to mediate resistance to colistin (51). In Taiwan in 2019, carbapenem-resistant *S. algae* carrying *bla*_OXA − 55_ with multiple genes encoding efflux pumps was detected in Asian hard clam (*Meretrix lusoria*) (52). In 2020 in Italy, OXA-55-like producing *S. algae* was found (53).

All the previous information demonstrated the presence of CROs in seafood and aquaculture. Even when bacteria in aquatic environments are mainly non-pathogenic, their occurrence highlights the relevance of the food production chain in the global spread of antibiotic-resistance genes. Moreover, it is crucial to consider the seafood market, where countries can consume a specific product imported from a separate region by another country, which could have different regulations for antimicrobial resistance surveillance in food. Canada is one example of this since shrimp consumed by Canadians are imported from Asian countries. However, carbapenemase genes have been detected in these products. Examples include the isolation of *Enterobacter cloacae* or *Enterobacter aerogenes* harboring *bla*_IMI − 1_, *bla*_IMI − 2_, or *bla*_NDM − 1_ genes in retail seafood (39, 54).

In addition, it is remarkably the high amount of antibiotics used and their multiple classes in aquaculture for prophylactic purposes or metaphylactic treatment. Quinolones, tetracyclines, amphenicols, and sulfonamides are the most commonly used classes (55); however, they also include aminoglycosides, antimycobacterial (rifampin), beta-lactams (aminopenicillins and cephalosporins), and polymyxins. This factor could promote selective pressure for the emergence of antimicrobial resistance and the selection of multidrug-resistant organisms among seafood animals. Indeed, global antimicrobial consumption is estimated to reach 13,600 tons by 2030 (55). Additionally, antibiotic residues are difficult to eliminate by water treatment plants and can be discharged into water flows, thus being a source of antimicrobial resistance genes.

Moreover, fish do not effectively metabolize antibiotics; thus, the active substance passes into the environment in the feces. Indeed, it has been suggested that ~70–80% of the antibiotics applied in aquaculture are dispersed into water systems (37), which might provide a selection and enrichment mechanism for resistant bacteria (22). Studies reporting the occurrence of carbapenem-resistant genes worldwide in seafood and aquaculture are summarized in Table 2.

**Table 2:** Occurrence of carbapenemase-encoding genes in seafood and aquaculture.

**Year**	**Animal origen**	**Country**	**Bacteria**	**Carbapenemase-encoding genes**	**References**
2013	Fish	Algeria	*A. baumannii*	*bla*_OXA − 23_	(34)
2014	Squid	Canada	*Pseudomonas fluorescens*-like	*bla*_VIM − 2_	(37)
2015	Squid, sea squirt, seafood medley, clam	Canada	*Stenotrophomonas maltophilia, Myroides odoratimimus, Stenotrophomonas* spp., and *P. putida*	*bla*_OXA − 48_	(36)
	Retail seafood	Originated from Korea and China			
2015	Shrimp	Canada	*E. cloacae*	*bla*_IMI − 1_	(38)
	Retail seafood			*bla*_NDM − 1_	
				*bla*_OXA − 1_	
2015	Shrimp	Canada	*E. aerogenes*	*bla*_IMI − 2_	(38)
	Retail seafood				
2016	Shrimp	Canada	*V. cholerae*	*bla*_VCC − 1_	(39)
	Retail seafood				
2016	Venus clam	Italy	*E. coli* ST10	*bla*_VIM − 1_	(40)
2016	Oyster	Tunisia	*A. baumannii*	*bla*_OXA − 23_	(47)
2016	Fish	Algeria	*A. baumannii*	*bla*_OXA − 23_	(34)
2016	Bivalves (oyster)	Tunisia	*A. baumannii*	*bla*_OXA − 23_	(46)
2018	Fish	India	*Enterobacteriaceae (K. pneumoniae, E. coli)*	*bla*_NDM − 1_	(40)
	Retail seafood			*bla*_NDM − 2_	
				*bla*_NDM − 5_	
2018	Shirmp	France	*V. parahaemolyticus*	*bla*_NDM − 1_	(48)
2018	Abalone	Taiwan	*S. algae*	*bla*_OXA − 55_	(51)
2019	Hard clam	Taiwan	*S. algae*	*bla*_OXA − 55_	(52)
2019	White shrimp	The Netherlands	*E. cloacae* complex	*bla*_IMI − 2_	(33)
	Retail seafood	Originated in India		*bla*_FLC − 2_	
2019	Shrimp	China	*V. alginolyticus*	*bla*_VIM − 1_	(31)
	Retail seafood		*V. parahaemolyticus*	*bla*_NDM − 1_	
			*V. vulnificus*	*bla*_NDM − 1_	
2019	Hard-shelled mussel	Korea	*Vibrio* spp.	*bla*_OXA_	(43)
	Retail seafood				
2020	Tilapia fish	Egypt	*E. coli*	*bla*_OXA − 48_	(50)
	Fish farm		*E. cloacae* complex	*bla*_NDM_	
			*K. pneumoniae*	*bla*_KPC_	
2020	Fish	Italy	*S. algae*	*bla*_OXA − 55_	(53)
	Aquaculture farms				
2020	Shrimp	China	*V. alginolyticus*	*bla*_VMB − 1_	(15)
	Retail seafood				
2021	Shrimp	China	*V. diabolicus*	*bla*_VMB − 2_	(32)
	Retail seafood				
2021	Shrimp	China	*V. alginolyticus*	*bla*_VAM − 1_	(17)

## 3. Occurrence of carbapenem-producing bacteria in terrestrial food-producing animals

Intensive farming has frequently been associated with the excessive use of antimicrobials and drug-resistant microorganisms isolated from food-producing animals that can be transmitted to humans via direct contact with animals or ingestion of derived food products (9). By 2030, global antimicrobial use from human, terrestrial, and aquatic food-producing animal sectors will reach 236,757 tons annually, with an estimated proportion of terrestrial food-producing animal use of 174,549 tons, representing 73.7% of the global consumption of antimicrobials (55). This intensive use of antibiotics creates selective pressure for the emergence of antimicrobial resistance among farmers and the environment. In addition, antibiotics continue to be used in livestock production as prophylaxis and animal growth support (9), which may lead to the emergence of antimicrobial resistance.

The transmission of antimicrobial resistance between food-producing animals and humans can occur via the food chain, by consuming food products contaminated with antimicrobial resistance genes or antimicrobial-resistant bacteria, through direct contact between humans and animals, or through shared environmental sources such as contaminated water (56, 57). In addition, resistance can be transmitted to livestock from environmental sources (i.e., hospital sewage, wastewater treatment plants contaminating water and soil, surface water flow, and wildlife) and biological vectors, such as flies and wild birds (21, 58). Moreover, livestock growth promotion antibiotics may interact with the animal gut microbiota and introduce increased variation in antimicrobial resistance genes (ARGs) in the gut (59), thus increasing their dispersion. Studies reporting the occurrence of carbapenem-resistant genes worldwide in terrestrial food-producing animals are summarized in Table 3.

**Table 3:** Occurrence of carbapenemase-encoding genes in terrestrial food-producing animals.

**Year**	**Country**	**Carbapenemase-encoding genes**	**Bacteria**	**Source**		**References**
2011	Germany	*bla*_VIM − 1_	*E. coli*	Pig	Pig farm	(60)
2011	Germany	*bla*_VIM − 1_	*S. enterica* subsp. *enterica* serovar Infantis	Pig	Pig farm	(61)
2013	China	*bla*_NDM_	*A. baumannii*	Pig	Lung sample	(62)
2015	China	*bla*_NDM − 5_	*E. coli*	Pig	Commercial pig farm	(63)
2016	US	*bla*_IMP − 27_	*E. coli, P. mirabilis, Morganella morganii, Providencia rettgeri, Proteus vulgaris, Enterobacter cancerogenus, Citrobacter braakii, E. cloacae, Citrobacter* spp., *Citrobacter farmeri, Citrobacter koseri*, and *Klebsiella oxytoca*	Environmental and fecal samples on pig farms	Pig farm (nursery rooms)	(57)
2016	Germany	*bla*_VIM − 1_	*Salmonella* Infantis	Pig	Sick piglet	(61)
2017	Germany and Italy	*bla*_OXA − 181_, *bla*_OXA − 48 − like_	*E. coli*	Pig	Fecal samples	(30)
2018	Italy	*bla*_OXA − 486_, *bla*_OXA − 396_, *bla*_OXA − 50_	*P. oryzihabitans, P. aeruginosa*	Pig	Slaughter	(9)
2022	Italy	*bla*_OXA − 181_	*E. coli*	Pig	Fattening	(64)
2012	China	*bla*_NDM − 1_	*Acinetobacter lwoffii*	Broiler	Poultry	(65)
2016	Egypt	*bla*_OXA − 48_, *bla*_KPC_, *bla*_NDM_	*K. pneumoniae*	Chicken	Broiler-poultry farming	(60)
2017	Egypt	*bla*_NDM − 1_, *bla*_NDM − 2_	*E. coli*	Retail chicken carcasses	Supermarkets, poultry slaughterhouses, and butcher shops	(66)
2020	China	*bla*_NDM − 5_, *bla*_NDM − 1_, *bla*_NDM − 9_, *bla*_NLM_	*Enterobacteriaceae* (*K. pneumoniae, E. coli*)*, Morganellaceae, Alcaligenes faecalis, P. putida*	Poultry farm	Chicken	(21)
2021	China	*bla*_NDM_, *bla*_OXA − 1_, *bla*_OXA − 10_	*P. mirabilis*	Chicken	Slaughterhouses/chicken farm	(67)
2021	China	*bla*_NDM − 1_	*P. mirabilis*	Broiler	Chicken	(68)
2015–2021	China	*bla*_NDM_	*E. coli*	Broiler farm	Chicken feces	(58)
2021	Egypt	*bla*_OXA − 1_, *bla*_KPC_, *bla*_NDM_	*P. mirabilis*	Ducks	Farm	(69)
2021	China	*bla*_NDM_, *bla*_VIM_, *bla*_KPC_, *bla*_OXA − like_		Broiler	Cooperative broiler feedlot	(70)
2023	China	*bla*_NDM_, *bla*_OXA_	*P. mirabilis, E. coli, K. pneumoniae*	Broiler	Fattening farm	(71)
2010	France	*bla*_OXA − 23_	*A. baumannii*	Cattle	Dairy farm	(72)
2016	US	*bla*_OXA − 497_	*A. baumannii*	Cattle	Dairy cattle	(73)
2016	Egypt	*bla*_OXA − 48_, *bla*_OXA − 181_, *bla*_OXA − 7_, *bla*_OXA − 10_	ESBL-producing *E. coli*	Cattle	Dairy cattle farms	(29)
2016	India	*bla*_NDM − 5_	*E. coli*	Cattle	Bovine	(74)
2016	Algeria	*bla*_NDM − 5_	*E. coli*	Cattle	Raw milk	(75)
2017	China	*bla*_NDM − 5_	*K. pneumoniae*	Cattle	Dairy cows	(76)
2017	Germany	*bla*_OXA − 23_	*Acinetobacter indicus*-like	Cattle	Calves	(77)
2018	US	*bla*_KPC − 2_	*K. pneumoniae*	Cattle	Beef cattle	(78)
2019	India	*bla*_VIM_	*E. coli*	Cattle	Calves	(79)
2019	Egypt	*bla*_KPC_, *bla*_OXA − 48_, *bla*_NDM_	*P. aeruginosa*	Buffaloes and cattle	Farm	(80)
2020	South Africa	*bla*_KPC_, *bla*_NDM_, *bla*_GES_, *bla*_OXA − 48_, *bla*_VIM_, *bla*_OXA − 23_	*E.coli, K. pneumoniae, P. mirabilis, Salmonella*	Cattle	Beef cattle	(81)
2022	Spain	*bla*_NDM − 1_	*E. coli*	Cattle	Dairy calves	(82)
2022	Italy	*bla*_OXA − 181_, *bla*_OXA − 48_	*E. coli*	Cattle	Bovine beef	(64)
2022	Pakistan	*bla*_OXA − 23_, *bla*_OXA − 51_, *bla*_NDM − 1_, *bla*_IMP_	*A. baumannii*	Cattle	Dairy cattle and beef cattle	(83)
2023	Tunisia	*bla*_OXA − 48_, *bla*_IMP_	ESBL-producing *E. coli*	Cattle	Diarrheic calves	(84)

### 3.1. Carbapenem resistance in swine

In pigs, carbapenem resistance has been observed in microorganisms from different bacterial species, including *E. coli, Salmonella, P. aeruginosa*, and *A. baumannii*. In 2011, VIM-1-producing *E. coli* ST88 and *Salmonella* Infantis harboring *bla*_VIM − 1_IncHI2 plasmids were reported in Germany (60, 61). ST88 was also previously identified among chickens, cattle, and humans in Germany. Moreover, class 1 integron harbored by an IncH12 plasmid was found in human strains (60). In addition, during the sampling period in the same year (2011), 35 isolates were positive for *bla*_VIM − 1_, indicating that carbapenemase-producing bacteria may persist in livestock farms. Another study reported VIM-1-producing *Salmonella* Infantis in Germany in 2017 (59). The encoded genes *bla*_VIM − 1_ and *bla*_NDM − 5_ have reported resistance to third-generation cephalosporins used in animal husbandry. Moreover, *Salmonella* Infantis is one of the leading causes of human salmonellosis in Europe and a zoonotic pathogen commonly transferred via contaminated food products (59). In 2017, Pulss et al. (30) reported a porcine *E. coli* isolated carrying OXA-181 carbapenemase and the coexistence of *mcr-1* (mobilized colistin resistance gene) and acquired carbapenemase gene *bla*_OXA − 48 − like_ on isolates originated from Italy farms. *bla*_OXA − 181_ gene was located on al IncX3 plasmid (pEcIHIT31346-OXA-181), which presented high nucleotide similarity >99% to previously published plasmids from human sources (plasmid pOXA181_14828 of an *E. coli* isolated from a human patient in China) and also carried *qnrS1* (plasmid-media quinolone resistance gene), thus providing evidence of the possible link between human- and animal-derived carbapenem resistance. In 2015, *bla*_IMP − 27_ was detected in *Proteus mirabilis, Morganella morganii, Providencia rettgeri, Proteus vulgaris, Enterobacter cancerogenus, Citrobacter braakii, Enterobacter cloacae, Citrobacter* spp., *Citrobacter farmeri, Citrobacter koseri*, and *Klebsiella oxytoca* in the United States, within an IncQ1 plasmid recovered from the nursery and farrowing barns of a swine production system (57). In 2013 in China, *A. baumannii* harboring *bla*_NDM − 1_ genes isolated from lung samples of pigs with pneumonia and sepsis were identified (62). Meropenem-resistant *P. aeruginosa* strains carrying *bla*_OXA − 486_, *bla*_OXA − 396_, *bla*_OXA − 50_, and *bla*_PAO_ were also found in Italy in 2018, as were meropenem-resistant isolates of *Pseudomonas oryzihabitans* and *P. aeruginosa* (9). Three other isolates of *P. aeruginosa* carrying *bla*_PAO_, *bla*_OXA − 50_, *bla*_OXA − 486_, and *bla*_OXA − 488_ were detected in animals reared on different farms (85). Interestingly, two isolates of *P. aeruginosa* ST938 carrying *bla*_PAO_ and *bla*_OXA − 396_ and the resistance genes to aminoglycosides [*aph(3*′*)-IIb*], fosfomycin (*fosA4)*, and chloramphenicol (*catB7*) were detected, one in a pig and another one in 83-year-old patients. However, no epidemiological links were demonstrable between the animal and the patient. Other sequence types found were ST274, ST782, and ST885. The presence of *bla*_OXA − 50_ is concerning because this variant confers a decreased susceptibility to ampicillin, ticarcillin, and meropenem. In addition, the OXA-50 family also comprises *bla*_OXA − 396_, *bla*_OXA − 486_, and *bla*_OXA − 488_ genes (9). *bla*_OXA48_-like contained no plasmid, and *bla*_OXA − 181_-carrying IncX plasmid has also been reported in *E. coli* isolated from Italian fattening pigs (64). In the study, the authors recovered samples from fattening pigs, cattle, and workers from slaughterhouses. Twenty-four isolates were positive for *bla*_OXA − 181_ and one for *bla*_OXA − 48_. The isolates presented high ST diversity within ST5229 with higher prevalence. Different plasmid replicons were present in the isolates, with IncX1 and IncX3, and IncF types being the most represented. OXA-48-producing isolates did not contain any plasmid replicon. Furthermore, the authors detected an OXA-181-producing *E. coli* belonging to ST410 isolated in two fecal samples from fattening pigs, described as a high-risk clone associated with *bla*_OXA − 181_ in human patients. Moreover, in China in 2017, *E. coli* harboring a carbapenem-resistance gene *bla*_NDM − 5_ and *mcr-1* were detected on IncX3 plasmid with a high degree of diversity of ST. ST156 was also previously reported in a Chinese hospital (63).

### 3.2. Carbapenem resistance in poultry

Among broiler farms, an increased prevalence of CRE has been shown, mainly in *E. coli, K. pneumoniae*, and *P. mirabilis*, harboring *bla*_NDM_ genes, with *bla*_NDM − 1_ and *bla*_NDM − 5_ being the most predominant in chicken farm environments. Specifically, farming presented a higher prevalence of CRO among the studies, mainly attributed to the heavy use of antimicrobials on farms, transportation activities, and inadequate farm disinfection and management. Similarly, *bla*_NDM_ genes are usually carried by the IncX3 plasmid, which is clinically significant because it contributes to disseminating various *bla*_NDM_ variant genes (86).

In 2010, carbapenem-resistant isolates were detected in eight chicken farms, six duck farms, and one pig slaughterhouse in China. One of these isolates, *Acinetobacter lwoffii*, was identified as positive for *bla*_NDM − 1_, particularly on a 270 kb plasmid (65). In 2021, NDM-producing *P. mirabilis* was reported in broiler chickens (68). The isolate harbors a plasmid named pSNYG35, a pPrY2001-like plasmid that shares high nucleotide identity with pHFK418-NDM and an NDM-1-encoding plasmid from clinical *P. mirabilis*. Recently, Su et al. (87) reported isolation rates of 3.57% for carbapenem-resistant *E. coli*, 10% for carbapenem-resistant *P. mirabilis*, and 3.03% for carbapenem-resistant *K. pneumoniae* in six broiler fattening farms in China. Among carbapenem-resistant isolates, six *E. coli* carried class I integron, one carried class II integron, four *P. mirabilis* carried class I or II integrons, and one *K. pneumoniae* carried class 1 integron. All of these isolates harbor *bla*_NDM_ and *bla*_OXA_ genes. In 2016 in Egypt, carbapenem-producing *K. pneumoniae* (CR-Kp) in broiler poultry farming was reported. The authors found that 42% of the isolates from poultry samples carried *bla*_NDM_ (11 isolates carried *bla*_KPC_, *bla*_OXA − 48_, and *bla*_NDM_; four isolates carried *bla*_KPC_, *bla*_NDM_ or *bla*_OXA − 48_ and one isolate carried *bla*_NDM_ alone) (70). Interestingly, the authors collected 49 fecal samples from workers and veterinarians working in the poultry farm; 56% of the samples were CR-Kp-positive, with all strains carrying the three carbapenemase genes *bla*_KPC_, *bla*_OXA − 48_, and *bla*_NDM_, and 5% of them displayed all the carbapenemase-encoding genes at the same time. Furthermore, the prevalence was higher in farm workers (67%) compared to veterinarians (33%), indicating that transmission could be facilitated by close contact between broilers and humans since the workers are in continuous contact with the animals and lived on the farm during the fattening program. However, the study did not compare clones or plasmids; non-genetic relationships between humans and chickens were found (70). Lately, in China in 2023, He et al. (58) observed the transmission of *bla*_NDM_-bearing plasmids of *E. coli* isolated from chickens between different farms and detected carbapenem-resistant isolates in farmlands, vegetable fields, and the environment of chicken farms. The authors performed a longitudinal study from 2015 to 2021 that demonstrated that the prevalence of *bla*_NDM_-positive clones and plasmids varied in different years, which suggested that new strains and plasmids are constantly being introduced into the farms. In 2020 in China, Zhai et al. (21) reported 279 NDM-producing bacteria, including *Enterobacteriaceae* (*K. pneumoniae, E. coli*), *Morganellaceae, Alcaligenes faecalis*, and *Pseudomonas putida*, with the variants NDM-5, NDM-1, and NDM-9 as well as a novel NDM-like-metallo-**β**-lactamase (NLM) within IncX3, IncA/C2, and IncFII as major *bla*_NDM_-carrying plasmid types among isolates. Moreover, they found the coexistence of *mcr-1* or *mcr-8* on *K. pneumoniae* positive for *bla*_NDM − 1_. The authors identified 14 sequence types among the *E. coli* isolates, with ST6751 being the most prevalent. ST6716, ST156, ST69, ST48, and ST10 were also found. STs 6751, 10, 125, and 746 were recovered from chicken and environmental samples (sewage trenches, corridor floors, drooping boards, nipple drinkers, and air). Most of the *K. pneumoniae* isolates were ST37, followed by ST3410 and ST726. Additionally, in China, Shi et al. (71) reported the presence of the resistance genes *bla*_NDM_, *bla*_VIM_, *bla*_KPC_, and *bla*_OXA − like_ on broiler, layer, and pig farms with a significative higher relative abundance on *bla*_OXA − like_ genes from 2016 to 2019. The authors detected a prevalence of 20–30% for *bla*_KPC_ and *bla*_VIM_ genes, respectively, and a prevalence of 75% for *bla*_NDM_, reflecting the great incidence of carbapenemase-producing genes in farming. Moreover, the study also found the coexistence of colistin resistance gene *mcr-1* and *bla*_NDM_ with pig and chicken farms displaying high prevalence. In 2020 in Egypt, 155 meropenem-resistant isolates were obtained from retail chicken meat, indicating that carbapenem-producing bacteria may enter the food chain. The study reported a single *K. pneumoniae* ST147 and a single *E. coli* ST648 producing NDM-1 and NDM-5. This last isolated carried also *bla*_OXA − 1_, *bla*_TEM − 1_, *bla*_CTX − M−3_, and *aac(6*′*)-Ib-cr*, while the *K. pneumoniae* harbored the *bla*_SHV − 1_, *bla*_CTX − M−15_, and *aac(6*′*)-Ib-cr* genes (71). NDM-producing ST648 *E. coli* has been reported in clinical isolates in India, the United Kingdom, and Australia. NDM-1-producing ST147 *K. pneumoniae* clone has been reported previously in Iraq, Oman, Tunisia, and Egypt from hospitalized patients (66). A study conducted in China in 2019 reported NDM-1-producing *P. mirabilis* recovered from commercial broilers in slaughterhouses (67). In 2021 in Egypt, *P. mirabilis* harbored *bla*_NDM − 1_, *bla*_OXA − 1_, and *bla*_KPC_ was isolated from ducks on a duck farm (69).

### 3.3. Carbapenem resistance in cattle

Carbapenem-resistant bacteria are rare in cattle. However, since 2012, more studies have reported CROs in cattle with OXA- and NDM-producing bacteria leading to carbapenem resistance. In 2010, nine OXA-23-producing *Acinetobacter* genomospecies 15TU were reported in France, with a Tn*2008* as a vehicle for the spread (72). In 2016 in the United States, a novel *bla*_OXA − 497_ gene was detected in *A. baumannii*, which is part of the OXA-51-like enzyme group and displays resistance to ertapenem; however, these enzymes are naturally occurring in *A. baumannii* (73, 88). In 2017, *bla*_OXA − 23_ harboring *Acinetobacter indicus*-like strains that displayed imipenem, meropenem, and doripenem resistance were isolated from nasal swabs of two calves in Germany. *bla*_OXA − 23_ was localized on the chromosome and surrounded by interrupted Tn*2008* transposon structures. In addition, genetic relatedness between bovine isolates and *Acinetobacter indicus* type strains A648^T^ and human clinical *A. indicus* isolates were found (77). In 2022, 27.7% of CRAB bacteria in Pakistan were reported to harbor *bla*_OXA − 23_ and *bla*_OXA − 51_ within 17 isolates carrying *bla*_IMP_ and one isolate carrying *bla*_NDM − 1_. The typical sequence types found were ST642 and the international clone ST2 (83). In Egypt in 2019, carbapenem-resistant *P. aeruginosa* (CRPA) was reported in buffaloes and cattle with a prevalence of 60 and 59% (50 total samples) within isolates harboring *bla*_KPC_, *bla*_OXA − 48_, and *bla*_NDM_. The authors also found carbapenem-resistance genes from drinking water within 67% of prevalence and from stool human samples within 80% of prevalence. Additionally, phylogenetic analysis showed that cattle and water sequences were in one cluster and more related to each other than to human isolates (80). Similarly, in Egypt in 2014, five *E. coli* carrying *bla*_OXA − 48_, and one *E. coli* carrying *bla*_OXA − 181_ were reported in dairy cattle (29). In South Africa, 28–42% of carbapenem resistance was found in isolates such as *E. coli, K. pneumoniae, P. mirabilis*, and *Salmonella* spp., carrying *bla*_KPC_, *bla*_NDM_, *bla*_OXA − 23_, *bla*_VIM_, *bla*_OXA − 48_ and *bla*_GES_ with different prevalence (81). In Italy, in 2021, the EU harmonized antimicrobial resistance (AMR) monitoring program reported that units of fattening pigs (21/301) and bovines (4/310) were positive for OXA-48-like *E. coli* (*n* = 24 OXA-181, *n* = OXA-48) (64). Most recently, in Tunisia, one isolate of ESBL-producing *E. coli* from calves with diarrhea carrying *bla*_OXA − 48_ and *bla*_IMP_ were reported (84).

Antimicrobial resistance mediated through NDM enzymes is present in cattle. In 2013 in India, *E. coli* harboring the *bla*_NDM − 5_ gene was detected in milk samples of dairy cattle suffering mastitis (74). NDM-5-producing *E. coli* isolates from raw milk collected in a dairy farm in Algeria and India in 2016 were again found (75, 89). *bla*_NDM − 1_ gene in *E. coli* isolated from cattle, carried in an IncC plasmid, was reported in 2022 in Spain. The IncC plasmid also carried genes for aminoglycoside, sulphonamide, and trimethoprim resistance (82). *K. pneumoniae* carrying *bla*_NDM − 5_ located on IncX3 plasmid was isolated from dairy cows in China in 2017 (76). The presence of IncX3 plasmid is highly relevant since it mediates the spread of genes encoding resistance to clinically relevant antibiotics. It has been reported to encode *qnrB7, qnrS*, *bla*_CTX − M−3_, *bla*_SHV − 12_, *bla*_KPC − 2_, *bla*_KPC − 3_, *bla*_NDM − 1_, *bla*_NDM − 4_, *bla*_NDM − 5_, *bla*_NDM − 7_, *bla*_NDM − 13_, *bla*_NDM − 17_, and *bla*_OXA − 181_ (86). The authors found that the *K*.*pneumoniaebla*_NDM − 5_ positive belonged to five STs, within ST1661 and ST2108, which were the most prevalent. The *bla*_NDM − 5_ gene was located on the ~46 kb IncX3 plasmid. The plasmid shared a similar genetic context and was nearly identical to the human *K. pneumoniae* plasmid (pNDM-MGR194) previously reported in India. Among beef cattle in the United States in 2018, isolates of *K. pneumoniae* carrying *bla*_KPC − 2_ from feces were detected in 72 samples (78). *bla*_VIM_ gene located on an Incl1 plasmid of a novel sequence type (ST 297) from *E. coli* was well-detected among calves from India in 2019 (79).

## 4. Occurrence of carbapenem resistance in wildlife

Antimicrobial resistance genes can colonize wild animals following contact with sewage, human waste, or animal manure (90). Human feces and manure runoff are the primary sources of AMR in wild animals, as intake water polluted with feces could directly or indirectly contaminate other animals and the surrounding environment. Moreover, AMR genes, such as carbapenemase genes, could originate from environmental bacteria, such as the OXA-48 family of enzymes, which occurs naturally in *Shewanella* spp., a genus that inhabits lake sediments (91, 92), and OXA-23 enzymes, which are almost entirely restricted to *A. baumannii* and originate from the environmental species *Acinetobacter radioresistens* (93). Chickens have been proposed as a source of carbapenemase-producing *Salmonella enterica* in livestock (85).

Several carbapenemase-producing bacteria have been reported worldwide in wildlife including the NDM, IMP, VIM, and OXA enzymes. In Germany, *Salmonella corvallis* carried *bla*_NDM − 1_ belonging to ST1541 isolated from black kites (*Milvus migrans*) were detected in 2013 (94). *bla*_NDM − 1_ gene was located in the IncA/C conjugative plasmid pRH-1738 and contained a fosfomycin-resistance gene (*fosA3* gen) (95). In 2016 in Australia, a high prevalence of *Salmonella* and IMP-4-producing *Enterobacteriaceae* was reported in silver gulls (96). The authors detected 120 carbapenem-resistant *Enterobacteriaceae* strains of 10 species, mainly *E. coli* carrying the *bla*_IMP − 4_, *bla*_IMP − 38_, and *bla*_IMP − 26_ genes, with a prevalence of 40% in the gulls. *bla*_IMP_ gene was carried by conjugative plasmids of variable sizes and diverse replicons, including HI2-N, HI2, A/C, A/C-Y, L/M, I1, and non-typeable plasmids. The authors showed that isolates from gulls have significant similarities with clinical isolates from Australia, suggesting the human origin of the isolates. In 2017, France reported 22 carbapenem-resistant VIM-1-producing *E. coli* in yellow-legged gull (*Larus michahellis*) isolated in 2012 (97). Interestingly, gulls live in close contact with humans; thus, wildlife may be an important transmission route of AMR. In 2018, carbapenem-producing *Enterobacteriaceae* isolates (two *E. coli* ST635 and one *K. pneumoniae* ST13) were reported in fecal samples from wild boars in Algeria, Africa. OXA-48-producing isolates were also resistant to amoxicillin, amoxicillin-clavulanate, tobramycin, ertapenem, and meropenem (98). In 2019, China reported a high frequency of carbapenemase producer isolates (350 isolates) in migratory birds (*Anser indicus, Phalacrocorax*, and *Larus ichthyaetus*), while 233 *Klebsiella* spp. and 2 *E. coli* isolates were NDM-5-carriers (99). In 2019 in Korea, zoonotic *Aeromonas* spp., resistant to imipenem and meropenem, were isolated from the nutria (*Myocastor coypus*). These isolates also carried the *cphA* gene (*Aeromonas hydrophila* gene) coding for a carbapenem-hydrolyzing metallo-**β**-lactamase (100). In the same year, in Algeria, carbapenemase-producing *K. pneumoniae* was reported in bat guano with OXA-48 and KPC-3 enzymes present in the isolates, as well as the resistance genes *bla*_TEM − 1_ (ampicillin resistance) and *aac(6*′*)-lb* (aminoglycoside resistance) (101). In 2019, 13 carbapenem-resistant *K. pneumoniae* were isolated from Barbary deer (*Cervus elaphus barbarus*) in Akfadou Forest in Algeria. The resistome of these isolates revealed the presence of *bla*_NDM − 1_, *bla*_CTX − M−15_, *bla*_SHV − 182_, *bla*_DHA − 1_, *bla*_OXA − 1_, *aac(3)-IIa, aac(3)-IId* (aminoglycoside resistance), *aac(6*′*)-Ib-cr* (aminoglycoside-fluoroquinolone resistance), *rmtC* (rRNA methyltransferase with high-level resistance to aminoglycosides), *sul1* (sulfonamides resistance), *qnrB9* (plasmid-mediated quinolone resistance), *fosA* (fosfomycin resistance), *tetA* (tetracycline resistance), *dfrA14* (trimethoprim resistance), *catA2, catB3* (chloramphenicol resistance), and *mphA* (macrolide-resistant phosphotransferase) genes. Five different plasmids, IncA/C2, IncFIA/(HI1), IncFIB(K), IncFII(K), and ColRNAI, were also found (102). Similarly, in 2020, carbapenem-resistant *K. pneumoniae* carried *bla*_OXA − 48_ on an incompatible group L/M plasmid found in seals (*Phoca vitulina*) (103), reflecting anthropogenic pollution as a source of AMR genes. In 2021, a genomic comparison was performed between *E. coli* carrying KPC-2 and *K. pneumoniae* containing KPC-3 isolated from gulls and humans in Alaska. The authors found varying levels of genetic similarity at discrete genetic loci with no evidence of direct transmission of *bla*_KPC_ between people and gulls; however, the conserved genetic elements surrounding *bla*_KPC_ suggest a possible exchange between species (104). In 2021 in India, five carbapenem-resistant *E. coli* were reported isolated from rescued sloth bear (*Melursus ursinus*). The isolates were positive for *bla*_NDM_ (60%, 3/5) carbapenemase gene and efflux pump-mediated carbapenem resistance (40%, 2/5), and co-harbor AMR genes *bla*_TEM − 1_, *bla*AmpC, *qnrA, qnrB, qnrS, tetA, tetB*, and *sulI* (105). In 2022, carbapenem-resistant *P. aeruginosa* strains were recovered from the feces of a red deer (*Cervus elaphus*) from Portugal, which resulted in a high-risk clone belonging to ST274 and co-harboring the genes *bla*_PAO_, *bla*_PDC − 24_, *bla*_OXA − 486_, *aph(3*′*)-lb* (aminoglycoside resistance), *fosA* (fosfomycin resistance), and *catB7* (chloramphenicol resistance), which are phenotypically resistant to imipenem and intermediate resistance to meropenem and doripenem (106). In 2022, a high diversity of carbapenem-resistance genes was found in wild birds sampled from Alaska, Chile, Spain, Ukraine, Turkey, and Pakistan. The authors found carbapenemase genes in diverse isolates, including *K. pneumoniae* carrying KPC, NDM, OXA, and VIM, as well as in hypervirulent CR-Kp isolates from gulls in Spain and Ukraine. Some isolates harbored antimicrobial resistance to up to 10 antibiotic classes, including colistin. OXA-48-producing *E. coli* in gulls in Alaska and Turkey and CRE from Chile and Spain also harbored colistin-resistance genes. Similarly, the authors found evidence of global temporal and spatial dissemination (107). In 2022 in Brazil, NDM-1-producing *E. coli* ST162 infecting a pygmy sperm whale (*Kogia breviceps*) was reported (108). Moreover, the resistome of the isolate carried genes conferring resistance to **β**-lactams (*bla*_NDM − 1_, *bla*_TEM − 1_, and *bla*_OXA − 1_), aminoglycosides [*aph(3*′*)-lb, aph(3*′*)-VI*], macrolide (*ermB, mdfA*, and *mphA*), rifamycin (*arr-3*), [*aac(6*′*)-Ib-cr*, and *qnrB6*], phenicols (*catB3* and *floR*), sulfonamide (*sul1* and *sul2*), and tetracycline (*tetA*), and plasmid replicons IncFIB and IncA/C2 were also detected. All the previous studies shown here demonstrate that wild animals are reservoirs of carbapenem-resistant bacteria. They provide a biological mechanism for spreading antibiotic-resistance genes and can facilitate their transmission to humans and livestock.

On the other hand, rivers and water flow are also an environment from the emergence of CROs. For example, *Pseudomonas fluorescens* was recovered from the Seine River (Paris, France) in 2010, which expressed PF-1, a novel Ambler class A carbapenemase (109). In 2022 in Poland, 301 carbapenem-resistant *Acinetobacter* strains were isolated from municipal wastewater and river water (110). In 2005, carbapenem-resistant bacteria were reported on water bodies in the United States (111, 112). In 2019, carbapenem-resistant bacteria on water bodies were isolated, including *Enterobacter asburiae, Aeromonas veronii, Cupriavidus gilardii, Pseudomonas*, and *Stenotrophomonas* spp. This study found that most strains were carbapenemase producers, and all the isolates of *Enterobacter asburiae* carried the *bla*_IMP − 2_ gene (111). Other studies have also reported the presence of carbapenem-resistant strains in seawater, stormwater, and surface runoff water at Costa locations in Sydney, Australia, in 2020 (113). Therefore, water environments are an important reservoir of bacteria resistant to carbapenems and other antibiotics, including bacteria carrying intrinsic and acquired carbapenemase genes.

## 5. Occurrence of carbapenem resistance in companion animals

Carpabenemase-producing Enterobacterales (CPE) and non-fermenting bacteria have also been reported in companion animals. As we pointed out before, carbapenems are not approved for veterinary use. The prescription is restricted to treating urinary tract infections and respiratory tract infections in dogs and cats originating from multidrug-resistant (MDR) *E. coli, K. pneumoniae*, and *P. aeruginosa* bacteria. Additionally, the treatment must be supported by a veterinarian specializing in infectious disease, and by a pharmacologist (114), even though the continuous evidence of carbapenem-producing bacteria in companion animals has been increasing.

Companion animals can acquire carbapenemase-producing bacteria through direct contact with colonized hosts and the through contaminated environments such as veterinary hospitals (115, 116). In this regard, the human-pet bond has favored the silent transmission of carbapenem-producing bacteria to companion animals by a reverse zoonotic route called zooanthroponosis (115–117). Indeed, in Finland in 2015, identically isolates from dogs (with a long history of recurrent *otitis externa* without carbapenem prescription) and human family members with NDM-5-producing multidrug-resistant ST167 *E. coli* were reported. In addition, the same family carried an identical extended-spectrum beta-lactamase (ESBL) CTX-M-group 9 *E. coli* ST69, indicating interspecies transmission (118). In 2018 in Brazil, six VIM-2 carbapenemase-producing *P. aeruginosa* ST233 isolates were recovered from an infected dog, its owner (with a history of hospitalization), and its domestic environment (sofa, balcony, and water cooler) (116). ST233 has been reported as an international high-risk clone associated with carbapenemase production with resistance to all antimicrobial drugs. It has generally been restricted to human hospital settings (119–121), suggesting a zooanthroponotic transmission of this clone after the patient's hospital discharge. More recently, in 2022 in Guangzhou, China, a large-scale investigation on the prevalence of *bla*_NDM_-positive *E. coli* isolates from companion animals and their healthcare providers in clinical veterinary settings revealed the clonal spread *bla*_NDM_-positive ST453 *E. coli* isolates between both species (122). In France in 2022, OXA-48-producing *K. pneumoniae* were isolated from companion animals (dogs, cats, horses, cattle, and birds) with 56.2% (59/105 isolates) of the isolates belonging to the human-associated MDR ST11, ST15, and ST307 lineages, suggesting that numerous human-associated clones could infect the animal host (123).

Among carbapenemases on companion animals, NDM-5 and OXA-48-like carbapenemases are the most frequently described enzymes, with *E. coli* and *K. pneumoniae* being the main carbapenem-producing Enterobacterales, along with the non-fermenting bacteria *A. baumannii* (124). OXA-48 has been identified in *Enterobacteriaceae* from dogs and cats in different countries, such as Germany (2013) and the United States (2009–2013) (27, 125), as well as in an ST38 *E. coli* isolated from fowl (*Gallus domesticus*) in 2015 in Lebanon (28). In 2012, in Belgium, two OXA-23-producing *Acinetobacter* spp. were detected in fecal samples from 20 hospitalized horses, both resistant to imipenem and presented resistance to tetracyclines, sulfonamides, trimethoprim, and gentamicin but were still susceptible to colistin (126).

On the other hand, the KPC enzyme has also been reported. In 2018 in Brazil, in *K. pneumoniae* and *E. coli* from dogs, the *bla*_KPC − 2_ gene was found in Tn*4401* transposons contained in IncN plasmids, which also carried *bla*_CTX − M−15_, and other clinically significant resistance determinants conferring resistance to aminoglycosides (*aadA5*), quinolines (*qnrS1*), macrolides [*mph(A)* and *erm(B*)], sulfonamides (*sul1*), tetracycline [*tet(B)*], and trimethoprim (*dfrA17*), and point of mutation conferring quinolone resistance (127). In Brazil 2021, the KPC-2-producing *K. pneumoniae* belonging to the high-risk international clone ST11/CG258 in a dog with urinary tract infection carrying the IncN plasmid assigned to ST15 was reported (128). The *bla*_KPC − 4_ gene was detected in 2016 in Ohio, US, in an IncHI2 plasmid in the context of the Tn*4401b* transposon in *Enterobacter xiangfangensis* isolated from a clinical dog sample with ST171, which has been responsible for major clusters of human CRE infections in the northeastern and upper-midwestern of the United States (129, 130). IMP-4 has been reported in *Salmonella enterica* serovar Typhimurium isolated from cats in Australia in 2016 (131). NDM-1 was isolated in 2013 in the United States from dogs and cats from *E. coli* that also carried *bla*_CTX − M−15_ and belonged to ST167 (132), as well as in China, with *Acinetobacter* species carrying *bla*_NDM − 1_ and *bla*_OXA − 23_ (133), and in Italy from *A. radioresistens* (134). Recently, in 2022 in China, five *bla*_NDM − 5_ harboring *E. coli* were reported in dogs and cats, all of them multidrogo resistant. The *bla*_NDM − 5_ gene was located on 46 kb IncX3 plasmids in the five strains. Additionally, one strais coharbored *bla*_NDM − 5_-encoding-IncX3 plasmid along with an *mcr-1*-IncX4 hybrid plasmid (135). OXA-48 has mainly been described in dogs, cats, and horses and mostly from infections such as urinary tract infections (UTIs) isolates from *E. coli, K. pneumoniae, E. cloacae*, and *K. oxytoca* (136, 137). VIM-1 and VIM-2 were also reported in dogs infected with *K. pneumoniae* and *P. aeruginosa* in Spain and Korea in 2016 and 2018, respectively (138, 139), as well as OXA-181-producing extra-intestinal pathogenic *E. coli* ST410 from a dog in Portugal in 2020 (140), OXA-23-mediated carbapenem-resistance *A. baumannii* ST2 from a cat (141), and OXA-66-producing *A. baumannii* isolated from cats (124, 142).

## 6. Transmission between animals and humans

Among transmission between animals and humans, few studies have investigated the evidence for established links between human- and animal-derived carbapenem resistance. In 2022, Shen et al. (143) reported 29,799 *E. coli* isolates recovered from patients at 30 hospitals in China, as well as 61 pig farms and 45 chicken farms in 2017. From human clinical isolates, 631 were defined as carbapenem-resistant *E. coli* (CREc, 2.1%) with 195 NDM-positive. For livestock production, *bla*_NDM_ was detected in 73.8% (*n* = 45) and 62.2% (*n* = 28) of pig and chicken farms, respectively. Furthermore, they found that human NDM-positive *E. coli* isolates shared 15 (*n* = 111), 11 *(n* = 90), and 10 (*n* = 96) STs with those from chickens, pigs, and flies, respectively. NDM-positive isolates belonging to ST167, ST206, ST10, and ST48 were recovered from all four origins. Furthermore, the authors found that large proportions of *bla*_NDM_ genes (>70%) were associated with IncX3 plasmid in both animals (pig, chicken, and fly isolates) and humans. The authors also predicted the origins of 463 NDM-positive isolates. They found that 19% (*n* = 24), 8.1% (*n* = 10), and 1.6% (*n* = 2) of chicken NDM-positive *E. coli* isolates (*n* = 123) were predicted to originate from humans, pigs, and flies, respectively. In contrast, 27.3% (27/99) of pig NDM-positive *E. coli* isolates were predicted to originate from humans. Similarly, 53.8% (*n* = 105) and 14.9% (*n* = 29) of human isolates were predicted to have originated from chickens and pigs. All fly-derived isolates (*n* = 46) were predicted to have originated from humans (*n* = 5, 10.9%), chickens (*n* = 22, 47.8%), and pigs (*n* = 19, 41.3%). These results indicated positive associations and transmission of CREc between animals and humans. Indeed, the authors hypothesize that “CREc first arose in clinical settings and was then introduced into livestock animals, which are favorable hosts for the persistence of CREc. This led to the circulation of CREc between humans and animals, either via the food chain or through environmental vectors”.

In 2019, Li et al. (144) sampled 12 villages in China used as pig production farms [using the household as a single surveillance unit (resident and their backyard animals, including farm and companion animals)] and two commercial pig farms near the villages. The authors collected flies, fecal samples from humans, pigs, chickens, cattle, goats, ducks, one donkey, dogs, and cats across the villages, and additional fecal samples from pigs and farm workers at the two commercial farms. They obtained 88 CREC isolates that contained the *bla*_NDM_ carbapenemase gene, 17 from humans, 44 from pigs, 12 from chickens, 12 from flies, two from dogs, and one from cattle. No CREC isolates were recovered from workers of pigs at the two nearest commercial pig farms. The authors detected *bla*_NDM − 5_, *bla*_NDM − 1_, and *bla*_NDM − 9_, with most of these *bla*_NDM_-genes likely located on IncX3-type plasmids. Indeed, the *bla*_NDM_-carrying regions/plasmid (IncX3) in CRE isolates from humans exhibited >99% nucleotide sequence identity to those in isolates from backyard animals and flies. MLST showed that six human CRE-NDM-positive isolates displayed ST48, ST10, ST1114, or ST6910 shared by animal isolates. ST48 was the most prevalent and was associated with isolates from pigs, humans, chickens, and flies. Furthermore, they found that two human isolates displayed only three single-nucleotide polymorphisms (SNPs) with two pig isolates from the same village. They also reported that CREC isolates from flies have human and dog origins, while chicken isolates had a predominant origin from pigs and dogs. In addition, the single cattle-derived isolate was clustered with the chicken isolates. Therefore, many CRE isolates from humans, backyard animals, and flies originated from hosts other than those included in the study.

In 2017, Wang et al. (145) recovered 245 CRE from poultry (chicken farms, slaughterhouses, and supermarkets), dogs, sewage, wild birds, flies, and farmers. The authors identified *bla*_NDM_ in 21.8% (*n* = 161) of the *E. coli* isolates, 7.4% on *K*. *pneumoniae*, and 3.9% in *E. cloacae*, with *bla*_NDM − 5_, *bla*_NDM − 9_, *bla*_NDM − 1_, and *bla*_NDM − 7_ variants. Importantly, 23% of CREC isolates were also positive for *mcr-1*. High rates of CREC were found in dogs' feces (82.4%), flies (25.8%), wild birds' nests (40%), and anal swabs of farmers (50%). The most prevalent STs among *bla*_NDM_-positive isolates were ST101, ST156, and ST746. Moreover, MLST analysis showed commonality between strains from chicken farms, slaughterhouses, supermarkets, and humans, typified by genotypes ST10 and ST156. The authors confirm the commonality of ST156 isolates among disparate samples by core-genome single-nucleotide polymorphism (SNP)-based phylogenetic analysis. Additionally, *bla*_NDM_-carrying contigs gave three main genomic backbone profiles. The type II backbone included the contigs from 84 isolates derived from chicken cloacae (*n* = 37), flies (*n* = 21), dog feces (*n* = 12), chicken meat from supermarkets (*n* = 5), sewage from the farm (*n* = 1), chicken caeca from a slaughterhouse (*n* = 1), feces from farmers (*n* = 3), swallows (*n* = 3), and sewage from a slaughterhouse (*n* = 1). Type II genomic backbone was found in 26 *E. coli* isolates and shares >99.9% nucleotide sequence identity with the corresponding region of a 46,253bp IncX3 plasmid pJEG027 from *K. pneumoniae* isolated from an Australian traveler who was repatriated to Sydney from Myanmar.

The studies above showed a positive association between livestock production and human CREC infections since they identified a close relationship between the genomic profile of carbapenem-resistant isolates from humans and animals. High similarities between isolates from different sources were found. However, the studies only focused on NDM-positive isolates and not on other carbapenemases-encoding genes. Further studies are needed to elucidate the link between humans and animals.

## 7. One health approach for antimicrobial resistance

Different strategies to combat antimicrobial resistance have been developed, including the One Health approach, the EU Harmonized AMR Monitoring Program conducted in Italy in 2021, the National Action Plan for Combating Antibiotic-Resistant Bacteria (CARB) by the US (2020–2025), and the implementation of antimicrobial risk assessment.

One Health approach is a term recognized in the EU in 2016 by the United Nations Political Declaration on Antimicrobial Resistance (AMR), which states that human health, animal health, and the environment are interconnected and that disease is transmitted from humans to animals and vice versa. Furthermore, the environment could be a potential source of new resistant microorganisms; therefore, AMR should be addressed in all scenarios (25, 146, 147).

Strictly, One Health is defined as “a collaborative, multisectoral, and trans-disciplinary approach—working at local, regional, national, and global levels—to achieve optimal health (and wellbeing) outcomes recognizing the interconnections between people, animals, plants and their shared environment” (146). Therefore, a multidisciplinary approach is required to prevent the spread and emergence of antimicrobial resistance. Antimicrobial resistance (AMR) surveillance using the One Health approach has been implemented in Europe, the UK, and the US to mitigate the crisis. However, the lack of implementation in most developing countries resulted in the underestimation of the burden of AMR on terrestrial and aquatic animals and the environment (147).

Improper management of antimicrobials, such as inadequate control of infection, use of antimicrobials as growth promoters (long-term, low-dose mass medication), prophylaxis in livestock, farmed fish in aquaculture systems, agricultural debris, environmental pollutants from sewage, pharmaceutical industry waste, manure runoff from farms, use of heavy metals, use of disinfectants, and migration of people and animals infected with resistant bacteria, facilitate the spread of resistance between humans and animals (25, 148). Consequently, the One Health approach is fundamental since it is a multidisciplinary approach that tries to prevent, predict, detect, and respond to AMR (25, 148).

Critical strategies for addressing AMR from the One Health perspective includes: (1) conduct a global campaign to raise awareness of antimicrobial resistance and the damage caused by the overuse and misuse of antibiotics, (2) improve hygiene measures and prevent the spread of infections (i.e., decrease missing of animals from different sources, stress of transport, unsanitary or crowded conditions), (3) reduce the use of antimicrobials in agriculture and their dissemination to the environment (including third-generation cephalosporins, fluoroquinolones, colistin, tetracyclines, and macrolides; i.e., growth promoters such as colistin has been banned in Europe, Canada, Denmark, United States, and other countries) (24), (4) improve global surveillance of drug resistance in order to understand and clarify the new mechanisms of resistance acquisition and predict future threats, (5) promote new and rapid clinical diagnoses, (6) promote the development and use of vaccines and alternatives to antibiotics (i.e., phage therapy, probiotics, antibodies, lysins, among others), (7) improve the number of studies in the field, (8) generated a global innovation fund for early-stage research on new treatments, (9) promote investment in new drugs and in the improvement of existing drugs, and finally, (10) build a global coalition for real action against AMR (25, 148, 149).

All previous studies have highlighted the urgent need to establish a One Health AMR surveillance system to understand the magnitude of the AMR problem, specifically the carbapenem-resistance problem, identify trends, and determine how all scenarios are linked and establish settings to content the widespread carbapenem-resistant organisms and genes. This approach requires the integration of human healthcare, livestock, aquaculture, and the environment, as well as other variety of disciplines and fields (149). Furthermore, the role of infections caused by antimicrobial-resistant organisms in wildlife may also have to be addressed, along with resistant organisms from aquatic environments, as they could possess intrinsic resistance and the possibility of being transmitted horizontally. In addition, carbapenem-resistant in companion animals has to take seriously since the human-pet bond might favor the silent transmission of clinically significant multidrug-resistant bacteria through zooanthroponosis (148).

Moreover, antimicrobial residues in fish products can persist in aquatic environments through excreta. For example, testing foodstuffs for carbapenem-resistant bacteria is not a legal requirement in any country; however, even a low prevalence of carbapenem-resistant genes has been detected in imported shrimp and salmon. In addition, studies have shown that aquaculture and terrestrial farms exhibit significant differences in drug consumption, with the aquaculture sector exhibiting the lowest. However, commensal bacterial flora can act as reservoirs of AMR genes, which may be transferred to microorganisms capable of causing human and animal diseases. Furthermore, it has been documented that animals excrete a significant percentage (75–90%) of antimicrobials without being metabolized and dispersed into the environment (145), which could be taken up by wild animals and function as a reservoir for antimicrobial resistance genes (149).

## 8. Discussion

The reports on carbapenem-resistant organisms published from seafood and aquaculture are still low. Most of these reports must include information on potential sources or transmission between humans, animals, and their environment. Similarly, some of the microorganisms found are of clinical importance. Some examples are carbapenem-producing *Vibrio alginolyticus*, which causes vibriosis, wound infection, and ear infection; *Vibrio cholerae*, which causes cholera; *Vibrio parahaemolyticus*, that cause acute, self-limiting gastroenteritis (150); *Shewanella algae*, a potential foodborne zoonotic agent in humans that causes necrotizing fasciitis, discitis, meningitis, biliary infection, pneumonia, and endocarditis (151); and *Enterobacter cloacae* complex that is common in nosocomial settings and capable of producing several infections such as pneumonia, urinary tract infections, and septicemia (152).

On livestock, carbapenem resistance has been observed in microorganisms from different bacterial species, critical in human settings, and associated with significant public health concerns worldwide, including *E. coli, Salmonella, P. mirabilis, P. aeruginosa*, and *A. baumannii*. In swine and poultry settings, VIM-1-producing *Salmonella* Infantis was found. This bacterium is a zoonotic pathogen commonly transferred via contaminated food products that have been implicated in human salmonellosis and foodborne outbreaks associated with egg and chicken meat (153). For instance, *A. baumannii* is an important opportunistic pathogen for hospital-acquired infections commonly associated with multi-drug resistance. The mortality rate of *A. baumannii* infection has been estimated to be over 50% (154). In livestock such as cattle and pigs, *A. baumannii* causes mastitis, pneumonia, and sepsis. In companion, animals cause urinary tract infections (155–157). Whether the presence of OXA-23-producing *A. baumannii* poses a substantial public health threat is unclear, but the presence of NDM-1 producers in *A. lwoffii and A. baumannii* isolated from poultry, swine, and cattle, which are clinically relevant to humans, is worrying. Similarly, NDM-producing *E. coli* and *K. pneumoniae*, isolated from poultry and cattle, are worrisome since they have been reported in clinical isolates worldwide.

The carbapenem-resistance determinants in wild animals need to be better understood. Wild animals may act as potential environmental reservoirs for bacterial resistance. VIM, NDM, OXA-48, and KPC-producing *K. pneumoniae* are the more frequent carbapenemases reported, followed by IMP, and NDM-producing *E. coli*. Contaminated food and water are the main routes of transmission of carbapenem-resistance bacteria to wild animals (158). However, anthropogenic pressure plays an essential role in the emergence of resistance, particularly in this setting. Interestingly, migrating birds (i.e., gulls) have been proposed to serve as a vehicle for disseminating carbapenem-resistance genes (159).

Among food-producing animals, the link between farming practices, animal health, carbapenem-resistant organisms' development and spread to farmers, and the presence of carbapenem-resistant organisms in foodstuffs requires much more investigation. Three studies positively associated livestock production with human CREC infections (143–145). These associations are mainly based on the observation that *bla*_NDM_-carrying IncX3 plasmid isolated from humans exhibited between 75 and 99% nucleotide sequence identity to those in isolates from other sources, including chicken, pigs, and fly isolates. Moreover, one article identified a close relationship between the core-genome sequences of NDM-positive *E. coli* from humans and animals. The source-tracing analysis revealed indistinct boundaries between human- and animal-derived NDM-positive *E. coli* (143). Several studies have found genetic similarities between carbapenem-producing bacteria from animal and human sources, including the detection of a porcine *E. coli* isolated carrying OXA-181 carbapenemase located on al IncX3 plasmid with high nucleotide similarity (99%) to previously published plasmid from human sources (30); *E. cloacae* IMI-1 positive isolated from clam and human-source *E. cloacae* ST373 isolate harboring *bla*_IMI − 1_ sharing >75% similarity (8), and the detection of varying levels of genetic similarity at discrete genetic loci between *E. coli* carrying KPC-2 and *K. pneumoniae* containing KPC-3 isolated from gulls and humans in Alaska (104).

In companion animals, carbapenem resistance has been reported. The enzymes NDM-5, VIM, KPC, OXA-48-like, and OXA-23 were detected in *E. coli, K. pneumoniae, P. aeruginosa*, and *A. baumannii* (124–142). In this setting, the evidence suggests that zooanthroponosis is the main route of transmission of carbapenem-producing bacteria from humans to companion animals, indicating a cross-species transmission (115–123). Remarkably, in carbapenem-resistant organisms isolated from all the sources presented here (food-producing animals, seafood, aquaculture, wildlife, and companion animals), NDM-1 enzymes are occurring. These enzymes can hydrolyze all β-lactam antibiotics and have a high potential for rapid dissemination, thus, may constitute a public health risk (159).

These examples demonstrated that direct anthropozoonotic or zooanthroponotic transmission might be possible for CRE. However, to estimate the public health relevance of this transmission, more studies are needed to elucidate the problem. The addition of high-throughput technology, such as whole-genome sequencing and next-generation sequencing (NGS), has been permitted to determine the genetic relationship among CRO from different species at gene, plasmid, and strain levels. Similarly, introducing discriminant analysis of principal components (DAPCs) is helpful for tracing carbapenem-producing strains' potential origins.

## 9. Conclusion

The data presented in this review confirm the widespread of carbapenemase-producing bacteria and encoding genes in food-producing animals, seafood, aquaculture, companion animals, and wildlife as a cause of representing a severe problem for human and animal health.

Several studies have shown genetic similarities between human and animal carbapenem-resistance isolates, thus, demonstrating the possible cross-species transmission. Nonetheless, epidemiologic and genotypic analysis studies are needed to understand better the dynamics of antimicrobial drug resistance transmission between humans, animals, and the environment. In addition, the presence of CROs in the food chain compromises food safety and security and increases the chance of cross-border transmission of these bacteria.

One Health approach can help to implement global monitoring programs and establish antimicrobial risk assessments for the zoonotic and environmental sectors to address AMR emergencies. It is essential to identify and share best practices and policies globally. Collaboration between governments is needed to address cross-border health threats of AMR.

## Author contributions

AG-B conceived and designed the idea for the manuscript. FR-C and AG-B wrote the manuscript. AG-B, FR-C, and FA-G reviewed the manuscript. All authors read and approved the final manuscript.

## References

[1] Nordmann P Dortet L Poirel L. Carbapenem resistance in *Enterobacteriaceae*: here is the storm! *Trends Mol Med* (2012) 18:263–72. 10.1016/j.molmed.2012.03.003 22480775

[2] Gupta N Limbago BM Patel JB Kallen AJ. Carbapenem-resistant *Enterobacteriaceae*: epidemiology and prevention. Clin Infect Dis. (2011) 53:60–7. 10.1093/cid/cir202 21653305

[3] Aurilio C Sansone P Barbarisi M Pota V Giaccari LG Coppolino F. Mechanisms of action of carbapenem resistance. Antibiotics. (2022) 11:421. 10.3390/antibiotics11030421 35326884 PMC8944602

[4] Codjoe FS Donkor ES. Carbapenem resistance: a review. Med Sci. (2017) 6. 10.3390/medsci6010001 29267233 PMC5872158

[5] Meletis G Protonotariou E Papadopoulou D Skoura L. Comment on: the carbapenemase menace: do dual mechanisms code for more resistance? Infect Control Hosp Epidemiol. (2016) 37:1392–4. 10.1017/ice.2016.197 27573218

[6] G.Taggar, Attiq Rheman M, Boerlin P, Diarra MS. Molecular epidemiology of carbapenemases in *Enterobacteriales* from humans, animals, food and the environment. Antibiotics. (2020) 9:693. 10.3390/antibiotics9100693 33066205 PMC7602032

[7] Anderson REV Boerlin P. Carbapenemase-producing *Enterobacteriaceae* in animals and methodologies for their detection. Can J Vet Res. (2020) 84:3–17. 31920216 PMC6921994

[8] Mangat CS Boyd D Janecko N Martz SL Desruisseau A Carpenter M. Characterization of VCC-1, a novel Ambler Class A carbapenemase from *Vibrio cholerae* isolated from imported retail shrimp sold in Canada. Antimicrob Agents Chemother. (2016) 60:1819–25. 10.1128/AAC.02812-15 26824956 PMC4775928

[9] Bonardi S Cabassi CS Manfreda G Parisi A Fiaccadori E Sabatino A. Survey on carbapenem-resistant bacteria in pigs at slaughter and comparison with human clinical isolates in Italy. Antibiotics. (2022) 11:777. 10.3390/antibiotics11060777 35740183 PMC9219774

[10] Walther-Rasmussen J Høiby N Class A. carbapenemases. J Antimicrob Chemother. (2007) 60:470–82. 10.1093/jac/dkm226 17595289

[11] Queenan AM Bush K. Carbapenemases: the versatile beta-lactamases. Clin Microbiol Rev. (2007) 20:440–58. 10.1128/CMR.00001-07 17630334 PMC1932750

[12] Hammoudi Halat D Ayoub Moubareck C. The current burden of carbapenemases: review of significant properties and dissemination among gram-negative bacteria. Antibiotics. (2020) 9:186. 10.3390/antibiotics9040186 32316342 PMC7235769

[13] Bonnin RA Jousset AB Emeraud C Oueslati S Dortet L Naas T. Genetic diversity, biochemical properties, and detection methods of minor carbapenemases in *Enterobacterales*. Front Med. (2021) 7:616490. 10.3389/fmed.2020.616490 33553210 PMC7855592

[14] Castanheira M Toleman MA Jones RN Schmidt FJ Walsh TR. Molecular characterization of a beta-lactamase gene, *bla*GIM-1, encoding a new subclass of metallo-beta-lactamase. Antimicrob Agents Chemother. (2004) 48:4654–61. 10.1128/AAC.48.12.4654-4661.2004 15561840 PMC529189

[15] Zheng Z Cheng Q Chan EW Chen S. Genetic and biochemical characterization of VMB-1, a novel metallo-β-Lactamase encoded by a conjugative, broad-host range IncC plasmid from *Vibrio* spp. Adv Biosyst. (2020) 4:e1900221. 10.1002/adbi.201900221 32293144

[16] Lee K Yum JH Yong D Lee HM Kim HD Docquier JD. Novel acquired metallo-beta-lactamase gene, *bla*SIM-1, in a class 1 integron from *Acinetobacter baumannii* clinical isolates from Korea. Antimicrob Agents Chemother. (2005) 49:4485–91. 10.1128/AAC.49.11.4485-4491.2005 16251286 PMC1280121

[17] Cheng Q Zheng Z Ye L Chen S. Identification of a novel metallo-β-lactamase, VAM-1, in a foodborne *Vibrio alginolyticus* isolate from China. Antimicrob Agents Chemother. (2021) 65:e0112921. 10.1128/AAC.01129-21 34424042 PMC8522725

[18] Zhao WH Hu Z-Q. Acquired metallo-**β**-lactamses and their genetic association with class 1 integrons and ISCR elements in Gram-negative bacteria. Fut Microbiol. (2015) 10. 10.2217/fmb.15.18 26000655

[19] Castanheira M Sader HS Farrell DJ Mendes RE Jones RN. Activity of ceftaroline-avibactam tested against Gram-negative organism populations, including strains expressing one or more β-lactamases and methicillin-resistant *Staphylococcus aureus* carrying various staphylococcal cassette chromosome mec types. Antimicrob Agents Chemother. (2012) 9:4779–85. 10.1128/AAC.00817-12 22733066 PMC3421892

[20] Hansen GT. Continuous evolution: perspective on the epidemiology of carbapenemase resistance among Enterobacterales and other Gram-negative bacteria. Infect Dis Ther. (2021) 10:75–92. 10.1007/s40121-020-00395-2 33492641 PMC7954928

[21] Zhai R Fu B Shi X Sun C Liu Z Wang S. Contaminated in-house environment contributes to the persistence and transmission of NDM-producing bacteria in a Chinese poultry farm. Environ Int. (2020) 139:105715. 10.1016/j.envint.2020.105715 32315891

[22] Nordmann P Cuzon G Naas T. The real threat of *Klebsiella pneumoniae* carbapenemase-producing bacteria. Lancet Infect Dis. (2009) 9:228–36. 10.1016/S1473-3099(09)70054-4 19324295

[23] van Duin D Doi Y. Outbreak of colistin-resistant, carbapenemase-producing *Klebsiella pneumoniae*: are we at the end of the road? J Clin Microbiol. (2015) 53:3116–7. 10.1128/JCM.01399-15 26202122 PMC4572557

[24] Shein AMS Wannigama DL Higgins PG Hurst C Abe S Hongsing P. High prevalence of *mgrB*-mediated colistin resistance among carbapenem-resistant *Klebsiella pneumoniae* is associated with biofilm formation, and can be overcome by colistin-EDTA combination therapy. Sci Rep. (2022) 12:12939. 10.1038/s41598-022-17083-5 35902639 PMC9334626

[25] McEwen SA Collignon PJ. Antimicrobial resistance: a one health perspective. Microbiol Spectr. (2018) 6. 10.1128/microbiolspec.ARBA-0009-2017 29600770 PMC11633550

[26] EFSA Panel on Biological Hazards (BIOHAZ). Scientific Opinion on Carbapenem resistance in food animal ecosystems. EFSA J. (2013) 11:3501. 10.2903/j.efsa.2013.3501

[27] Liu X Thungrat K Boothe DM. Occurrence of OXA-48 carbapenemase and other β-lactamase genes in ESBL-producing multidrug resistant *Escherichia coli* from dogs and cats in the United States, 2009-2013. Front Microbiol. (2016) 7:1057. 10.3389/fmicb.2016.01057 27462301 PMC4939299

[28] Al Bayssari C Olaitan AO Dabboussi F Hamze M Rolain JM. Emergence of OXA-48-producing *Escherichia coli* clone ST38 in fowl. Antimicrob Agents Chemother. (2015) 59:745–6. 10.1128/AAC.03552-14 25348536 PMC4291434

[29] Braun SD Ahmed MF El-Adawy H Hotzel H Engelmann I Weiss D. Surveillance of extended-spectrum beta-lactamase-producing *Escherichia coli* in dairy cattle farms in the Nile Delta, Egypt. Front Microbiol. (2016) 7:1020. 10.3389/fmicb.2016.01020 27458435 PMC4931819

[30] Pulss S Semmler T Prenger-Berninghoff E Bauerfeind R Ewers C. First report of an *Escherichia coli* strain from swine carrying an OXA-181 carbapenemase and the colistin resistance determinant MCR-1. Int J Antimicrob Agents. (2017) 50:232–6. 10.1016/j.ijantimicag.2017.03.014 28666753

[31] Zheng Z Ye L Chan EW Chen S. Identification and characterization of a conjugative *bla*VIM-1-bearing plasmid in *Vibrio alginolyticus* of food origin. J Antimicrob Chemother. (2019) 74:1842–7. 10.1093/jac/dkz140 30993329

[32] Liu M Zhang W Peng K Wang Z Li R. Identification of a novel plasmid-mediated carbapenemase-encoding gene*, bla*VMB-2, in *Vibrio diabolicus*. Antimicrob Agents Chemother. (2021) 65:e0020621. 10.1128/AAC.00206-21 34097496 PMC8284434

[33] Brouwer MSM Tehrani HME Rapallini M Geurts Y Kant A Harders F. Novel carbapenemases FLC-1 and IMI-2 encoded by an *Enterobacter cloacae* complex esolated from food products. Antimicrob Agents Chemother. (2019) 63:e02338–18. 10.1128/AAC.02338-18 30910900 PMC6535546

[34] Brahmi S Touati A Cadiere A Djahmi N Pantel A Sotto A. First description of two sequence Type 2 *Acinetobacter baumannii* isolates carrying OXA-23 Carbapenemase in *Pagellus acarne* fished from the Mediterranean Sea near Bejaia, Algeria. Antimicrob Agents Chemother. (2016) 60:2513–5. 10.1128/AAC.02384-15 26787693 PMC4808192

[35] Vieira RH Carvalho EM Carvalho FC Silva CM Sousa OV Rodrigues DP. Antimicrobial susceptibility of *Escherichia coli* isolated from shrimp (*Litopenaeus vannamei*) and pond environment in northeastern Brazil. J Environ Sci Health B. (2010) 45:198–203. 10.1080/03601231003613526 20390951

[36] Morrison BJ Rubin JE. Carbapenemase producing bacteria in the food supply escaping detection. PLoS ONE. (2015) 10:e0126717. 10.1371/journal.pone.0126717 25966303 PMC4429064

[37] Rubin JE Ekanayake S Fernando C. Carbapenemase-producing organism in food, 2014. Emerg Infect Dis. (2014) 20:1264–5. 10.3201/eid2007.140534 24960459 PMC4073846

[38] Janecko N Martz SL Avery BP Daignault D Desruisseau A Boyd D. Carbapenem-resistant *Enterobacter* spp. in retail seafood imported from Southeast Asia to Canada. Emerg Infect Dis. (2016) 22:1675–7. 10.3201/eid2209.160305 27533255 PMC4994362

[39] Roschanski N Guenther S Vu TTT Fischer J Semmler T Huehn S. VIM-1 carbapenemase-producing *Escherichia coli* isolated from retail seafood, Germany 2016. Euro Surveill. (2017) 22:17-00032. 10.2807/1560-7917.ES.2017.22.43.17-00032 29090680 PMC5718389

[40] Das UN Singh AS Lekshmi M Nayak BB Kumar S. Characterization of *bla*(NDM)-harboring, multidrug-resistant *Enterobacteriaceae* isolated from seafood. Environ Sci Pollut Res Int. (2019) 26:2455–63. 10.1007/s11356-018-3759-3 30471059

[41] Kim DH Rajapaksha LG Gunasekara CW Wimalasena SH Pathirana HN Kim SR. Phylogenetic relationships and antibiotic resistance of *Vibrio parahaemolyticus* isolates related to acute hepatopancreatic necrosis disease in Korea. Aquaculture. (2021) 545:737253. 10.1016/j.aquaculture.2021.737253

[42] Dahanayake PS Hossain S Wickramanayake M Heo GJ. Prevalence of virulence and extended-spectrum beta-lactamase (ESBL) genes harbouring *Vibrio* spp. isolated from cockles (*Tegillarca granosa*) marketed in Korea. Lett Appl Microbiol. (2020) 71:61–9. 10.1111/lam.13232 31602660

[43] Hossain S Wickramanayake M Dahanayake PS Heo GJ. Occurrence of virulence and extended-spectrum beta-lactamase leterminants in *Vibrio* spp. Isolated from marketed hard-shelled mussel (*Mytilus coruscus*). Microb Drug Resist. (2020) 26:391–401. 10.1089/mdr.2019.0131 31596685

[44] Ottaviani D Medici L Talevi G Napoleoni M Serratore P Zavatta E. Molecular characterization and drug susceptibility of non-O1/O139 *V. cholerae* strains of seafood, environmental and clinical origin, Italy. Food Microbiol. (2018) 72:82–8. 10.1016/j.fm.2017.11.011 29407408

[45] Serratore P Zavatta E Fiocchi E Serafini E Serraino A Giacometti F. Preliminary study on the antimicrobial susceptibility pattern related to the genotype of *Vibrio vulnificus* strains isolated in the north-western Adriatic Sea coastal area. Ital J Food Saf. (2017) 6:6843. 10.4081/ijfs.2017.6843 29564231 PMC5850071

[46] Mani Y Mansour W Mammeri H Denamur E Saras E Boujâafar N Bouallègue O J-Madec Y Haenni M KPC-3-producing ST167 Escherichia coli from mussels bought at a retail market in Tunisia. J Antimicrob Chemotherapy. (2017) 72:2403–4. 10.1093/jac/dkx124 28472479

[47] Mani Y Mansour W Lupo A Saras E Bouallègue O Madec JY. Spread of *bla*CTX-M-15-producing *Enterobacteriaceae* and OXA-23-producing *Acinetobacter baumannii* sequence type 2 in Tunisian seafood. Antimicrob Agents Chemother. (2018) 62:e00727–18. 10.1128/AAC.00727-18 29967023 PMC6125568

[48] Briet A Helsens N Delannoy S Debuiche S Brisabois A Midelet G. NDM-1-producing *Vibrio parahaemolyticus* isolated from imported seafood. J Antimicrob Chemother. (2018) 73:2578–9. 10.1093/jac/dky200 29796645

[49] Sperling L Alter T Huehn S. Prevalence and antimicrobial resistance of *Vibrio* spp. in retail and farm shrimps in Ecuador. J Food Prot. (2015) 78:2089–92. 10.4315/0362-028X.JFP-15-160 26555534

[50] Hamza D Dorgham S Ismael E Siel-Moez A Elhariri M Elhelw R. Emergence of β-lactamase- and carbapenemase-producing *Enterobacteriaceae* at integrated fish farms. Antimicrob Resist Infect Control. (2020) 9. 10.1186/s13756-020-00736-3 32430083 PMC7236517

[51] Huang YT Cheng JF Chen SY Hong YK Wu ZY Liu PY. Draft genome sequence of carbapenem-resistant *Shewanella algae* strain AC isolated from small abalone (*Haliotis diversicolor*). J Glob Antimicrob Resist. (2018) 14:65–7. 10.1016/j.jgar.2018.06.005 29933118

[52] Lee YH Tung KC Cheng JF Wu ZY Chen SY Hong YK. Genomic characterization of carbapenem-resistant Shewanella algae isolated from Asian hard clam (*Meretrix lusoria*). Aquaculture. (2019) 500:300–4. 10.1016/j.aquaculture.2018.10.028

[53] Zago V Veschetti L Patuzzo C Malerba G MLleo M. Shewanella algae and *Vibrio* spp. strains isolated in Italian aquaculture farms are reservoirs of antibiotic resistant genes that might constitute a risk for human health. Mar Pollut Bull. (2020) 154:111057. 10.1016/j.marpolbul.2020.111057 32174504

[54] Loest D Uhland FC Young KM Li XZ Mulvey MR Reid-Smith R. Carbapenem-resistant *Escherichia coli* from shrimp and salmon available for purchase by consumers in Canada: a risk profile using the Codex framework. Epidemiol Infect. (2022) 150:e148. 10.1017/S0950268822001030 35968840 PMC9386791

[55] Schar D Klein EY Laxminarayan R Gilbert M Van Boeckel TP. Global trends in antimicrobial use in aquaculture. Sci Rep. (2020) 10:21878. 10.1038/s41598-020-78849-3 33318576 PMC7736322

[56] Mollenkopf DF Kleinhenz KE Funk JA Gebreyes WA Wittum TE. Salmonella enterica and *Escherichia coli* harboring *bla*CMY in retail beef and pork products. Foodborne Pathog Dis. (2011) 8:333–6. 10.1089/fpd.2010.0701 21034230

[57] Mollenkopf DF Stull JW Mathys DA Bowman AS Feicht SM Grooters SV. Carbapenemase-producing *Enterobacteriaceae* recovered from the environment of a swine farrow-to-finish operation in the United States. Antimicrob Agents Chemother. (2017) 61:e01298-16. 10.1128/AAC.01298-16 27919894 PMC5278694

[58] He W Gao M Lv L Wang J Cai Z Bai Y. Persistence and molecular epidemiology of *bla*(NDM)-positive Gram-negative bacteria in three broiler farms: A longitudinal study (2015-2021). J Hazard Mater. (2023) 446:130725. 10.1016/j.jhazmat.2023.130725 36630875

[59] Borowiak M Szabo I Baumann B Junker E Hammerl JA Kaesbohrer A. VIM-1-producing Salmonella Infantis isolated from swine and minced pork meat in Germany. J Antimicrob Chemotherapy. (2017) 72:2131–3. 10.1093/jac/dkx101 28369508

[60] Fischer J San José M Roschanski N Schmoger S Baumann B Irrgang A. Spread and persistence of VIM-1 Carbapenemase-producing *Enterobacteriaceae* in three German swine farms in 2011 and 2012. Vet Microbiol. (2017) 200:118–23. 10.1016/j.vetmic.2016.04.026 27234907

[61] Fischer J Rodríguez I Schmoger S Friese A Roesler U Helmuth R. Salmonella enterica subsp. enterica producing VIM-1 carbapenemase isolated from livestock farms. J Antimicrob Chemother. (2013) 68:478–80. 10.1093/jac/dks393 23034713

[62] Zhang WJ Lu Z Schwarz S Zhang RM Wang XM Si W. Complete sequence of the *bla*NDM-1-carrying plasmid pNDM-AB from *Acinetobacter baumannii* of food animal origin. J Antimicrob Chemother. (2013) 68:1681–2. 10.1093/jac/dkt066 23449827

[63] Kong LH Lei CW Ma SZ Jiang W Liu BH Wang YX. Various sequence types of *Escherichia coli* isolates coharboring *bla*NDM-5 and *mcr-1* genes from a commercial swine farm in China. Antimicrob Agents Chemother. (2017) 61:e02167–16. 10.1128/AAC.02167-16 27993847 PMC5328568

[64] Carfora V Diaconu EL Ianzano A Di Matteo P Amoruso R Dell'Aira E. The hazard of carbapenemase OXA-181-producing *Escherichia coli* spreading in pig and veal calf holdings in Italy in the genomics era: Risk of spill over and spill back between humans and animals. Front Microbiol. (2022) 13:1016895. 10.3389/fmicb.2022.1016895 36466661 PMC9712188

[65] Wang Y Wu C Zhang Q Qi J Liu H Wang Y. Identification of New Delhi metallo-β-lactamase 1 in *Acinetobacter lwoffii* of food animal origin. PLoS ONE. (2012) 7:e37152. 10.1371/journal.pone.0037152 22629360 PMC3356367

[66] Sadek M Poirel L Nordmann P Nariya H Shimamoto T Shimamoto T. Genetic characterisation of NDM-1 and NDM-5-producing Enterobacterales from retail chicken meat in Egypt. J Glob Antimicrob Resist. (2020) 23:70–1. 10.1016/j.jgar.2020.07.031 32889140

[67] Zhu X Zhang Y Shen Z Xia L Wang J Zhao L. Characterization of NDM-1-Producing Carbapenemase in *Proteus mirabilis* among Broilers in China. Microorganisms. (2021) 9:2443. 10.3390/microorganisms9122443 34946044 PMC8707091

[68] Xie X Zhang J Wang HN Lei CW. Whole genome sequence of a New Delhi metallo-β-lactamase 1-producing *Proteus mirabilis* isolate SNYG35 from broiler chicken in China. J Glob Antimicrob Resist. (2021) 24:266–9. 10.1016/j.jgar.2020.12.023 33476836

[69] Algammal AM Hashem HR Alfifi KJ Hetta HF Sheraba NS Ramadan H. atpD gene sequencing, multidrug resistance traits, virulence-determinants, and antimicrobial resistance genes of emerging XDR and MDR-*Proteus mirabilis. Sci Rep*. (2021) 11:9476. 10.1038/s41598-021-88861-w 33947875 PMC8096940

[70] Hamza E Dorgham SM Hamza DA. Carbapenemase-producing *Klebsiella pneumoniae* in broiler poultry farming in Egypt. J Glob Antimicrob Resist. (2016) 7:8–10. 10.1016/j.jgar.2016.06.004 27530998

[71] Shi X Li Y Yang Y Shen Z Cai C Wang Y. High prevalence and persistence of carbapenem and colistin resistance in livestock farm environments in China. J Hazard Mater. (2021) 406:124298. 10.1016/j.jhazmat.2021.125951 33168321

[72] Poirel L Berçot B Millemann Y Bonnin RA Pannaux G Nordmann P. Carbapenemase-producing *Acinetobacter* spp. in Cattle, France. Emerg Infect Dis. (2012) 18:523–5. 10.3201/eid1803.111330 22377480 PMC3309584

[73] Webb HE Bugarel M den Bakker HC Nightingale KK Granier SA Scott HM. Carbapenem-resistant bacteria recovered from faeces of dairy cattle in the High Plains Region of the USA. PLoS ONE. (2016) 11:e0147363. 10.1371/journal.pone.0147363 26824353 PMC4732617

[74] Ghatak S Singha A Sen A Guha C Ahuja A Bhattacharjee U. Detection of New Delhi metallo-beta-lactamse and extended-spectrum beta-lactamse genes in *Escherichia coli* isolated from mastitic milk samples. Transbound Emerg Dis. (2013) 60:385–9. 10.1111/tbed.12119 23870003

[75] Yaici L Haenni M Saras E Boudehouche W Touati A Madec JY. blaNDM-5-carrying IncX3 plasmid in *Escherichia coli* ST1284 isolated from raw milk collected in a dairy farm in Algeria. J Antimicrob Chemother. (2016) 71:2671–2. 10.1093/jac/dkw160 27165785

[76] He T Wang Y Sun L Pang M Zhang L Wang R. Occurrence and characterization of *bla*NDM-5-positive *Klebsiella pneumoniae* isolates from dairy cows in Jiangsu, China. J Antimicrob Chemother. (2017) 72:90–4. 10.1093/jac/dkw357 27621177

[77] Klotz P Göttig S Leidner U Semmler T Scheufen S Ewers C. Carbapenem-resistance and pathogenicity of bovine *Acinetobacter indicus*-like isolates. PLoS ONE. (2017) 12:e0171986. 10.1371/journal.pone.0171986 28207789 PMC5313175

[78] Vikram A Schmidt JW. Functional bla(KPC-2) Sequences are present in US beef cattle feces regardless of antibiotic use. Foodborne Pathog Dis. (2018) 15:444–8. 10.1089/fpd.2017.2406 29672169

[79] Murugan MS Sinha DK Vinodh Kumar OR Yadav AK Pruthvishree BS Vadhana P. Epidemiology of carbapenem-resistant *Escherichia coli* and first report of *bla*VIM carbapenemases gene in calves from India. Epidemiol Infect. (2019) 147:e159. 10.1017/S0950268819000463 31063112 PMC6518490

[80] Elshafiee EA Nader SM Dorgham SM Hamza DA. Carbapenem-resistant *Pseudomonas aeruginosa* originating from farm animals and people in Egypt. J Vet Res. (2019) 63:333–7. 10.2478/jvetres-2019-0049 31572812 PMC6749737

[81] Tshitshi L Manganyi MC Montso PK Mbewe M Ateba CN. Extended spectrum beta-Lactamase-resistant determinants among carbapenem-resistant *Enterobacteriaceae* from beef cattle in the North West Province, South Africa: a critical assessment of their possible public health implications. Antibiotics. (2020) 9:820. 10.3390/antibiotics9110820 33213050 PMC7698526

[82] Tello M Oporto B Lavín JL Ocejo M Hurtado A. Characterization of a carbapenem-resistant *Escherichia coli* from dairy cattle harbouring *bla*NDM-1 in an IncC plasmid. J Antimicrob Chemother. (2022) 77:843–5. 10.1093/jac/dkab455 34907439 PMC8865002

[83] Alam M Rasool MH Khan I Khurshid M Aslam B. Multilocus sequence typing of carbapenem-resistant *Acinetobacter baumannii* isolates harboring *bla*OXA-23 and *bla*IMP in cattle from Punjab, Pakistan. Microb Drug Resist. (2022) 28:997–1002. 10.1089/mdr.2022.0083 35985003

[84] Ben Haj Yahia A Tayh G Landolsi S Maamar E Galai N Landoulsi Z„ et al. First report of OXA-48 and IMP genes among extended-spectrum beta-lactamase-producing *Escherichia coli* isolates from diarrheic calves in Tunisia. Microb Drug Resist. (2023) 29:150–62. 10.1089/mdr.2022.0129 36695709

[85] Girlich D Naas T Nordmann P. Biochemical characterization of the naturally occurring oxacillinase OXA-50 of *Pseudomonas aeruginosa*. Antimicrob Agents Chemother. (2004) 48:2043–8. 10.1128/AAC.48.6.2043-2048.2004 15155197 PMC415580

[86] Liakopoulos A van der Goot J Bossers A Betts J Brouwer MSM Kant A. Genomic and functional characterisation of IncX3 plasmids encoding *bla*SHV-12 in *Escherichia coli* from human and animal origin. Sci Rep. (2018) 8:7674. 10.1038/s41598-018-26073-5 29769695 PMC5955891

[87] Su Y Xin L Zhang F Peng C Li Z Liu C. Drug resistance analysis of three types of avian-origin carbapenem-resistant *Enterobacteriaceae* in Shandong Province, China. Poult Sci. (2023) 102:102483. 10.1016/j.psj.2023.102483 36682131 PMC9876955

[88] Evans BA Amyes SG. OXA β-lactamases. Clin Microbiol Rev. (2014) 27:241–63. 10.1128/CMR.00117-13 24696435 PMC3993105

[89] Purkait D Ahuja A Bhattacharjee U Singha A Rhetso K Dey TK. Molecular characterization and computational modelling of New Delhi metallo-β-lactamase-5 from an *Escherichia coli* Isolate (KOEC3) of bovine origin. Indian J Microbiol. (2016) 56:182–9. 10.1007/s12088-016-0569-5 27570310 PMC4984578

[90] Pesapane R Ponder M Alexander KA. Tracking pathogen transmission at the human-wildlife interface: banded mongoose and *Escherichia coli*. Ecohealth. (2013) 10:115–28. 10.1007/s10393-013-0838-2 23612855

[91] Poirel L Héritier C Nordmann P. Chromosome-encoded ambler class D beta-lactamase of *Shewanella oneidensis* as a progenitor of carbapenem-hydrolyzing oxacillinase. Antimicrob Agents Chemother. (2004) 48:348–51. 10.1128/AAC.48.1.348-351.2004 14693565 PMC310178

[92] Potron A Poirel L Nordmann P. Origin of OXA-181, an emerging carbapenem-hydrolyzing oxacillinase, as a chromosomal gene in *Shewanella xiamenensis*. Antimicrob Agents Chemother. (2011) 55:4405–7. 10.1128/AAC.00681-11 21746953 PMC3165332

[93] Poirel L Figueiredo S Cattoir V Carattoli A Nordmann P. Acinetobacter radioresistens as a silent source of carbapenem resistance for *Acinetobacter* spp. Antimicrob Agents Chemother. (2008) 52:1252–6. 10.1128/AAC.01304-07 18195058 PMC2292503

[94] Fischer J Schmoger S Jahn S Helmuth R Guerra B. NDM-1 carbapenemase-producing *Salmonella enterica* subsp. enterica serovar Corvallis isolated from a wild bird in Germany. J Antimicrob Chemother. (2013) 68:2954–6. 10.1093/jac/dkt260 23818284

[95] Qin S Fu Y Zhang Q Qi H Wen JG Xu H. High incidence and endemic spread of NDM-1-positive *Enterobacteriaceae* in Henan Province, China. Antimicrob Agents Chemother. (2014) 58:4275–82. 10.1128/AAC.02813-13 24777095 PMC4136005

[96] Dolejska M Masarikova M Dobiasova H Jamborova I Karpiskova R Havlicek M. High prevalence of *Salmonella* and IMP-4-producing *Enterobacteriaceae* in the silver gull on Five Islands, Australia. J Antimicrob Chemother. (2016) 71:63–70. 10.1093/jac/dkv306 26472769 PMC4681372

[97] Vittecoq M Laurens C Brazier L Durand P Elguero E Arnal A. VIM-1 carbapenemase-producing *Escherichia coli* in gulls from southern France. Ecol Evol. (2017) 7:1224–32. 10.1002/ece3.2707 28303191 PMC5305998

[98] Bachiri T Bakour S Lalaoui R Belkebla N Allouache M Rolain JM. Occurrence of carbapenemase-producing *Enterobacteriaceae* isolates in the wildlife: first report of OXA-48 in wild boars in Algeria. Microb Drug Resist. (2018) 24:337–45. 10.1089/mdr.2016.0323 28799835

[99] Liao X Yang RS Xia J Chen L Zhang R Fang LX. High colonization rate of a novel carbapenem-resistant *Klebsiella* lineage among migratory birds at Qinghai Lake, China. J Antimicrob Chemotherapy. (2019) 74:2895–903. 10.1093/jac/dkz268 31340044

[100] Lim SR Lee D-H Park SY Lee S Kim HY Lee M-S. Wild Nutria (*Myocastor coypus*) is a potential reservoir of carbapenem-resistant and zoonotic *Aeromonas* spp. in Korea. Microorganisms. (2019) 7:224. 10.3390/microorganisms7080224 31366125 PMC6723217

[101] Gharout-Sait A Touati A Ahmim M Brasme L Guillard T Agsous A de Champs C. Occurrence of carbapenemase-producing *Klebsiella pneumoniae* in bat guano. Microbial Drug Resist. (2019) 25:1057–62. 10.1089/mdr.2018.0471 31021173

[102] Mairi A Barraud O Muggeo A de Champs C Touati A. Genomic analysis of a multidrug-resistant *Klebsiella pneumoniae* ST11 strain recovered from barbary deer (*Cervus elaphus barbarus*) in Akfadou forest, Algeria. J Global Antimicrob Resist. (2019) 22:300–4. 10.1016/j.jgar.2020.04.027 32380242

[103] Duff JP AbuOun M Bexton S Rogers J Turton J Woodford N. Resistance to carbapenems and other antibiotics in *Klebsiella pneumoniae* found in seals indicates anthropogenic pollution. Vet Rec. (2020) 187:154. 10.1136/vr.105440 32327551

[104] Ahlstrom CA Frick A Pongratz C Spink K Xavier C Bonnedahl J. Genomic comparison of carbapenem-resistant *Enterobacteriaceae* from humans and gulls in Alaska. J Glob Antimicrob Resist. (2021) 25:23–5. 10.1016/j.jgar.2021.02.028 33667702

[105] VinodhKumar OR Karikalan M Ilayaraja S Sha AA Singh BR Sinha DL. Multi-drug resistant (MDR), extended spectrum beta-lactamase (ESBL) producing and carbapenem resistant *Escherichia coli* in rescued Sloth bears (*Melursus ursinus*), India. Vet Res Commun. (2021) 45:163–70. 10.1007/s11259-021-09794-3 34041662

[106] Torres RT Cunha MV Ferreira H Fonseca C Palmeira JD A. high-risk carbapenem-resistant *Pseudomonas aeruginosa* clone detected in red deer (*Cervus elaphus*) from Portugal. Sci Total Environ. (2022) 829:154699. 10.1016/j.scitotenv.2022.154699 35318052

[107] Ahlstrom CA Woksepp H Sandegren L Mohsin M Hasan B Muzyka D. Genomically diverse carbapenem resistant *Enterobacteriaceae* from wild birds provide insight into global patterns of spatiotemporal dissemination. Sci Total Environ. (2022) 824:153632. 10.1016/j.scitotenv.2022.153632 35124031

[108] Sellera FP Cardoso B Fuentes-Castillo D Esposito F Sano E Fontana H. Genomic analysis of a highly virulent NDM-1-producing *Escherichia coli* ST162 infecting a pygmy sperm whale (Kogia breviceps) in South America. Front Microbiol. (2022) 13:915375. 10.3389/fmicb.2022.915375 35755998 PMC9231830

[109] Girlich D Poirel L Nordmann P. Novel ambler class A carbapenem-hydrolyzing beta-lactamase from a *Pseudomonas fluorescens* isolate from the Seine River, Paris, France. Antimicrob Agents Chemother. (2010) 54:328–32. 10.1128/AAC.00961-09 19901091 PMC2798510

[110] Hubeny J Korzeniewska E Buta-Hubeny M Zieliński W Rolbiecki D Harnisz M. Characterization of carbapenem resistance in environmental samples and *Acinetobacter* spp. isolates from wastewater and river water in Poland. Sci Total Environ. (2022) 822:153437. 10.1016/j.scitotenv.2022.153437 35122847

[111] Harmon DE Miranda OA McCarley A Eshaghian M Carlson N Ruiz C. Prevalence and characterization of carbapenem-resistant bacteria in water bodies in the Los Angeles-Southern California area. Microbiol Open. (2019) 8:e00692. 10.1002/mbo3.692 29987921 PMC6460273

[112] Aubron C Poirel L Ash RJ Nordmann P. Carbapenemase-producing *Enterobacteriaceae*, US rivers. Emerg Infect Dis. (2005) 11:260–4. 10.3201/eid1102.030684 15752444 PMC3320444

[113] Dewi D Götz B Thomas T. Diversity and genetic basis for carbapenem resistance in a coastal marine environment. Appl Environ Microbiol. (2020) 86. 10.1128/AEM.02939-19 32198174 PMC7205498

[114] Sellera FP Da Silva LCBA Lincopan N. Rapid spread of critical priority carbapenemase-producing pathogens in companion animals: a One Health challenge for a post-pandemic world. J Antimicrob Chemotherapy. (2021) 76:2225–9. 10.1093/jac/dkab169 34109407

[115] Sellera FP Lincopan N. Zooanthroponotic transmission of high-risk multi-drug-resistant pathogens: a neglected public health issue. J Infect Public Health. (2019) 12:294–5. 10.1016/j.jiph.2018.12.013 30609965

[116] Fernandes MR Sellera FP Moura Q Carvalho MP Rosato PN Cerdeira L. Zooanthroponotic transmission of drug-resistant *Pseudomonas aeruginosa*, Brazil. Emerg Infect Dis. (2018) 24:1160–2. 10.3201/eid2406.180335 29774849 PMC6004847

[117] Smith A Wayne AS Fellman CL Rosenbaum MH. Usage patterns of carbapenem antimicrobials in dogs and cats at a veterinary tertiary care hospital. J Vet Intern Med. (2019) 33:1677–85. 10.1111/jvim.15522 31119803 PMC6639476

[118] Grönthal T Österblad M Eklund M Jalava J Nykäsenoja S Pekkanen K. Sharing more than friendship – transmission of NDM-5 ST167 and CTX-M-9 ST69 *Escherichia coli* between dogs and humans in a family, Finland 2015. Euro Surveill. (2018) 23:1700497. 10.2807/1560-7917.ES.2018.23.27.1700497 29991384 PMC6152158

[119] Zafer MM Al-Agamy MH El-Mahallawy HA Amin MA Ashour EDS. Dissemination of VIM-2 producing *Pseudomonas aeruginosa* ST233 at tertiary care hospitals in Egypt. BMC Infect Dis. (2015) 15:122. 10.1186/s12879-015-0861-8 25880997 PMC4396152

[120] Wright LL Turton JF Livermore DM Hopkins KL Woodford N. Dominance of international ‘high-risk clones' among metallo-β-lactamase producing *Pseudomonas aeruginosa* in the UK. J Antimicrob Chemother. (2015) 70:103–10. 10.1093/jac/dku339 25182064

[121] Sader HS Reis AO Silbert S Gales AC. IMPs, VIMs and SPMs: the diversity of metallo-β-lactamases produced by carbapenem-resistant *Pseudomonas aeruginosa* in a Brazilian hospital. Clin Microbiol Infect. (2005) 11:73–6. 10.1111/j.1469-0691.2004.01031.x 15649310

[122] Wang MG Fang C Liu KD Wang LL Sun RY Zhang RM. Transmission and molecular characteristics of *bla*NDM-producing *Escherichia coli* between companion animals and their healthcare providers in Guangzhou, China. J Antimicrob Chemother. (2022) 77:351–5. 10.1093/jac/dkab382 34726693

[123] Garcia-Fierro R Drapeau A Dazas M Saras E Rodrigues C Brisse S. Comparative phylogenomics of ESBL-, AmpC- and carbapenemase-producing Klebsiella pneumoniae originating from companion animals and humans. J Antimicrob Chemotherapy. (2022) 77:1263–71. 10.1093/jac/dkac041 35224624 PMC9047677

[124] Silva JMD Menezes J Marques C Pomba CF. Companion animals-an overlooked and misdiagnosed reservoir of carbapenem resistance. Antibiotics. (2022) 11:533. 10.3390/antibiotics11040533 35453284 PMC9032395

[125] Stolle I Prenger-Berninghoff E Stamm I Scheufen S Hassdenteufel E Guenther S. Emergence of OXA-48 carbapenemase-producing *Escherichia coli* and *Klebsiella pneumoniae* in dogs. J Antimicrob Chemother. (2013) 68:2802–8. 10.1093/jac/dkt259 23833179

[126] Smet A Boyen F Pasmans F Butaye P Martens A Nemec A. OXA-23-producing *Acinetobacter* species from horses: a public health hazard? J Antimicrob Chemother. (2012) 67:3009–10. 10.1093/jac/dks311 22872446

[127] Sellera FP Fernandes MR Ruiz R Falleiros ACM Rodrigues FP Cerdeira L. Identification of KPC-2-producing *Escherichia coli* in a companion animal: a new challenge for veterinary clinicians. J Antimicrob Chemother. (2018) 73:2259–61. 10.1093/jac/dky173 29800301

[128] Sellera FP Fuga B Fontana H Esposito F Cardoso B Konno S. Detection of IncN-pST15 one-health plasmid harbouring *bla*KPC-2 in a hypermucoviscous *Klebsiella pneumoniae* CG258 isolated from an infected dog, Brazil. Transbound Emerg Dis. (2021) 68:3083–8. 10.1111/tbed.14006 33507616 PMC9290030

[129] Gomez-Simmonds A Hu Y Sullivan SB Wang Z Whittier S Uhlemann AC. Evidence from a New York City hospital of rising incidence of genetically diverse carbapenem-resistant *Enterobacter cloacae* and dominance of ST171, 2007-14. J Antimicrob Chemother. (2016) 71:2351–3. 10.1093/jac/dkw132 27118776 PMC4954924

[130] Daniels JB Chen L Grooters SV Mollenkopf DF Mathys DA Pancholi P. Enterobacter cloacae complex Sequence Type 171 Isolates Expressing KPC-4 Carbapenemase Recovered from Canine Patients in Ohio. Antimicrob Agents Chemother. (2018) 62:e01161-18. 10.1128/AAC.01161-18 30249699 PMC6256759

[131] Abraham S O'Dea M Trott DJ Abraham RJ Hughes D Pang S. Isolation and plasmid characterization of carbapenemase (IMP-4) producing *Salmonella enterica* Typhimurium from cats. Sci Rep. (2016) 6:35527. 10.1038/srep35527 27767038 PMC5073282

[132] Shaheen BW Nayak R Boothe DM. Emergence of a New Delhi metallo-β-lactamase (NDM-1)-encoding gene in clinical *Escherichia coli* isolates recovered from companion animals in the United States. Antimicrob Agents Chemother. (2013) 57:2902–3. 10.1128/AAC.02028-12 23587948 PMC3716147

[133] Cui L Lei L Lv Y Zhang R Liu X Li M. bla(NDM-1)-producing multidrug-resistant *Escherichia coli* isolated from a companion dog in China. J Glob Antimicrob Resist. (2018) 13:24–7. 10.1016/j.jgar.2017.10.021 29102775

[134] Gentilini F Turba ME Pasquali F Mion D Romagnoli N Zambon E. Hospitalized pets as a source of carbapenem-resistance. Front Microbiol. (2018) 9:2872. 10.3389/fmicb.2018.02872 30574124 PMC6291488

[135] Kuang X Yang R Ye X Sun J Liao X Liu Y. NDM-5-Producing *Escherichia coli* co-harboring *mcr-1* gene in companion animals in China. Animals. (2022) 12:1310. 10.3390/ani12101310 35625156 PMC9137672

[136] Pulss S Stolle I Stamm I Leidner U Heydel C Semmler T. Multispecies and clonal dissemination of OXA-48 carbapenemase in *Enterobacteriaceae* from companion animals in Germany, 2009-2016. Front Microbiol. (2018) 9:1265. 10.3389/fmicb.2018.01265 29963026 PMC6010547

[137] Yousfi M Touati A Muggeo A Mira B Asma B Brasme L. Clonal dissemination of OXA-48-producing *Enterobacter cloacae* isolates from companion animals in Algeria. J Glob Antimicrob Resist. (2018) 12:187–91. 10.1016/j.jgar.2017.10.007 29042339

[138] González-Torralba A Oteo J Asenjo A Bautista V Fuentes E Alós JI. Survey of carbapenemase-producing *Enterobacteriaceae* in companion dogs in Madrid, Spain. Antimicrob Agents Chemother. (2016) 60:2499–501. 10.1128/AAC.02383-15 26824947 PMC4808160

[139] Hyun JE Chung TH Hwang CY. Identification of VIM-2 metallo-β-lactamase-producing *Pseudomonas aeruginosa* isolated from dogs with pyoderma and otitis in Korea. Vet Dermatol. (2018) 29:186–e68. 10.1111/vde.12534 29575185

[140] Brilhante M Menezes J Belas A Feudi C Schwarz S Pomba C. OXA-181-Producing extraintestinal pathogenic *Escherichia coli* Sequence Type 410 isolated from a dog in Portugal. Antimicrob Agents Chemother. (2020) 64:e02298-19. 10.1128/AAC.02298-19 31964797 PMC7179305

[141] Pomba C Endimiani A Rossano A Saial D Couto N Perreten V. First report of OXA-23-mediated carbapenem resistance in sequence type 2 multidrug-resistant *Acinetobacter baumannii* associated with urinary tract infection in a cat. Antimicrob Agents Chemother. (2014) 58:1267–8. 10.1128/AAC.02527-13 24295971 PMC3910809

[142] Ewers C Klotz P Leidner U Stamm I Prenger-Berninghoff E Göttig S. OXA-23 and IS*Aba1*-OXA-66 class D β-lactamases in *Acinetobacter baumannii* isolates from companion animals. Int J Antimicrob Agents. (2017) 49:37–44. 10.1016/j.ijantimicag.2016.09.033 27890443

[143] Shen Y Hu F Wang Y Yin D Yang L Chen Y. Transmission of carbapenem resistance between human and animal NDM-positive *Escherichia coli* strains. Engineering. (2022) 15:24–33. 10.1016/j.eng.2021.07.03035456850

[144] Li J Bi Z Ma S Chen B Cai C He J. Inter-host transmission of carbapenemase-producing *Escherichia coli* among humans and backyard animals. Environ Health Perspect. (2019) 127:107009. 10.1289/EHP5251 31642700 PMC6910777

[145] Wang Y Zhang R Li J Wu Z Yin W Schwarz S. Comprehensive resistome analysis reveals the prevalence of NDM and MCR-1 in Chinese poultry production. Nat Microbiol. (2017) 6:16260. 10.1038/nmicrobiol.2016.260 28165472

[146] One Health High-Level Expert Panel (OHHLEP) Adisasmito WB Almuhairi S Behravesh CB Bilivogui P Bukachi SA . (One Health: A new definition for a sustainable and healthy future. PLoS Pathog. (2022) 18:e1010537. 10.1371/journal.ppat.1010537 35737670 PMC9223325

[147] Das S. The crisis of carbapenemase-mediated carbapenem resistance across the human-animal-environmental interface in India. Infect Dis Now. (2023) 53:104628. 10.1016/j.idnow.2022.09.023 36241158

[148] Velazquez-Meza ME Galarde-López M Carrillo-Quiróz B Alpuche-Aranda CM. Antimicrobial resistance: One Health approach. Vet World. (2022) 15:743–9. 10.14202/vetworld.2022.743-749 35497962 PMC9047147

[149] Ferri M Ranucci E Romagnoli P Giaccone V. Antimicrobial resistance: a global emerging threat to public health systems. Crit Rev Food Sci Nutr. (2017) 57:2857–76. 10.1080/10408398.2015.1077192 26464037

[150] Ottaviani D Leoni F Serram Serracca L Decastelli L Rocchegiani E Masini L. Nontoxigenic *Vibrio parahemolyticus* strains causing acute gastroenteritis. J Clin Microbiol. (2012) 50:4141–3. 10.1128/JCM.01993-12 23052317 PMC3502970

[151] Bauer MJ Stone-Garza KK Croom D Andreoli C Woodson P Graf PC-F. Shewanella algae infections in United States naval special warfare trainees. Open Forum Infect Dis. (2019) 6:ofz442 10.1093/ofid/ofz442 31696143 PMC6824531

[152] Annavajhala MK Gomez-Simmonds A Uhlemann A-C. (2019). Multidrug-resistant enterobacter cloacae complex emerging as a global, diversifying threat. Front. Microbiol. 10:44. 10.3389/fmicb.2019.00044 30766518 PMC6365427

[153] Pillai SD. Source tracing in Thailand following contamination of dog chew toys with Salmonella. In:Hoorfar J, editor. Woodhead Publishing Series in Food Science, Technology and Nutrition, Case Studies in Food Safety and Authenticity. Woodhead Publishing. p. 123–9. 10.1533/9780857096937.2.123

[154] Bulens SN Yi SH Walters MS Jacob JT Bower C Reno J. Carbapenem-nonsusceptible *Acinetobacter baumannii*, 8 US metropolitan areas, 2012–2015. Emerg Infect Dis. (2018) 24:727–34. 10.3201/eid2404.171461 29553339 PMC5875254

[155] Müller S Janssen T Wieler LH. Multidrug resistant Acinetobacter baumannii in veterinary medicine - emergence of an underestimated pathogen? BMTW. (2014) 127:435–46. 25872253

[156] van der Kolk JH Endimiani A Graubner C Gerber V Perreten V. Acinetobacter in Veterinary Medicine with emphasis on *A. baumannii*. J Glob Antimicrob Resist. (2018) 16:59–71. 10.1016/j.jgar.2018.08.011 30144636

[157] Wareth G Neubauer H Sprague LD. Acinetobacter baumannii – a neglected pathogen in veterinary and environmental health in Germany. Vet Res Commun. (2019) 43:1–6. 10.1007/s11259-018-9742-0 30591981

[158] Cole D Drum DJ Stalknecht DE White DG Lee MD Ayers S. Free-living Canada geese and antimicrobial resistance. Emerg Infect Dis. (2005) 11:935–8. 10.3201/eid1106.040717 15963291 PMC3367595

[159] Radhouani H Silva N Poeta P Torres C Correia S Igrejas G. Potential impact of antimicrobial resistance in wildlife, environment, human health. Front Microbiol. (2014) 5:23. 10.3389/fmicb.2014.00023 24550896 PMC3913889

